# *Psiadia punctulata* major flavonoids alleviate exaggerated vasoconstriction produced by advanced glycation end products

**DOI:** 10.1371/journal.pone.0222101

**Published:** 2019-09-06

**Authors:** Hossam M. Abdallah, Esraa M. Zakaria, Ali M. El-Halawany, Gamal A. Mohamed, Martin K. Safo, Hany M. El-Bassossy

**Affiliations:** 1 Department of Natural Products and Alternative Medicine, Faculty of Pharmacy, King Abdulaziz University, Jeddah, Saudi Arabia; 2 Department of Pharmacognosy, Faculty of Pharmacy, Cairo University, Cairo, Egypt; 3 Department of Pharmacology, Faculty of Pharmacy, Zagazig University, Zagazig, Egypt; 4 Department of Pharmacognosy, Faculty of Pharmacy, Al-Azhar University, Assuit Branch, Assuit, Egypt; 5 Department of Medicinal Chemistry, Institute for Structural Biology, Drug Discovery and Development, School of Pharmacy, Virginia Commonwealth University, Richmond, Virginia, United States of America; Max Delbruck Centrum fur Molekulare Medizin Berlin Buch, GERMANY

## Abstract

Exaggerated vasoconstriction plays important roles in vascular complication in aging and many diseases like diabetes. Here, we investigated the protective effect of *Psiadia punctulata* (PP) on advanced glycation end products (AGEs)-induced aggravated vasoconstriction. The effect of total methanol extract of PP leaves (PPT) on AGE-induced vascular injury was studied through bioassay-guided fractionation procedures in order to find the bioactive fraction and isolate the bioactive compounds. Vascular reactivity was studied using the isolated artery technique by adding cumulative concentrations of phenylephrine (PE) or acetyl choline (ACh). In addition, the antiglycating effect, as well as the effect on AGEs intermediates dityrosine and N`-formylkynurenine and their radical scavenging activity were measured. The results showed that PPT alleviated the AGEs-induced aggravated vasoconstriction in a concentration-dependent manner. The bioassay guided fractionation procedures suggested the chloroform fraction (Fr I) to be responsible for the activity. Chemical investigation of this fraction resulted in isolation of four major bioactive compounds that were identified as: umuhengerin (**1**), gardenin (**2**), luteolin-3`,4`-dimethyl ether (**3**), and 5,3`-dihydroxy-6,7,4`,5`-tetramethoxyflavone (**4**). The four compounds alleviated the exaggerated vasoconstriction in a dose dependent manner. In search for their mechanism of action, we observed that PPT, Fr. I and the isolated compounds did not improve the impaired vasodilation associated with AGEs exposure. PPT, Fr. I and the isolated compounds **1**–**4** inhibited AGEs formation and their protein oxidation intermediates. Furthermore, PPT, Fr. I and the isolated compounds **1**–**4** showed weak radical scavenging activity with compound **4** as the most potent. In conclusion, PPT appears to protect against AGEs-induced exaggerated vasoconstriction through antiglycation and radical scavenging activities.

## Introduction

Advanced glycation end products (AGEs) are emerging concern in the field of medical research owing to their contribution to many disease states. AGEs are a group of cross-linked derivatives that are formed irreversibly in serum and tissues via non-enzymatic reaction between reducing sugars and free amino groups of proteins, lipids, or nucleic acids [1]. Hyperglycemia, oxidative stress and food rich in proteins and fats, all are sources of AGEs [2]. When excessively high levels of AGEs reach tissues or serum, they can become pathogenic, causing various pathologies, including diabetic complications and cardiovascular diseases [3]. The pathologic effects of AGEs are related to their ability to promote oxidative stress and inflammation through receptor-dependent pathways [4, 5]. AGEs also bind with intra- and extracellular proteins, altering their structure and functions [4, 5]. In the vasculature, AGEs have deleterious effects, particularly on the endothelium. Endothelial cells participate in blood vessel formation, coagulation, fibrinolysis, regulation of vascular tone as well as in inflammatory reactions [6]. Studies show that AGEs impair endothelial function through activation of inflammatory cells [7], increasing permeability and subendothelial lipid entry [8], depletion of intracellular calcium stores [9], modification of intracellular matrix proteins, suppression of the endothelial NOS and promotion of oxidative stress [1].

In recent years, there has been increasing interest of bioactive compounds from plant sources owing to their efficacy, safety, relative availability and low cost. Finding natural bioactive compounds with anti-glycation properties provides future approaches for preventing AGEs-related complications. Some studies have shown plant-isolated substances to have AGE-inhibitory effects [10–12].

*Psiadia*, a genus within the sunflower family found in Africa, Asia and the Mascarene Islands have been used traditionally for their medicinal properties [13]. For example, the leaves and roots of *Psiadia punctulata* Vatke (PP) are used as analgesics, expectorant, antimicrobial and antiparasitic remedies [14]. Phytochemical investigations of PP have proposed a number of biologically active compounds including diterpenes, flavonoids, phenylpropanoids, coumarins and terpenoids [13, 15].

This study aims to investigate the potential protective effect of PP on AGE-induced aggravated vasoconstriction response in rat aorta, and through bio-guided approach, isolate and identify the bioactive compounds and assess their mechanisms of action.

## Material and methods

### Drugs and chemicals

Bovine serum albumin (BSA), aminoguanidine (AG), methylglyoxal (MG), acetylcholine (ACh), phenylephrine (PE), Nω-Nitro-L-argininemethyl ester hydrochloride (L-NAME), and diphenyl-2-picrylhydrazyl (DPPH) were all purchased from Sigma–Aldrich, Dorset, UK. Ultrapure deionized water was used as solvent except for the natural compounds and DPPH, which were dissolved in dimethyl sulfoxide (DMSO) in a concentration that did not exceed 0.1% in the reaction media. All other solvents and chemicals used were of analytical grade.

### General experimental procedures

UV spectra of the isolated compounds were detected on a Hitachi-300 spectrophotometer (Kyoto, Japan). NMR spectra of isolated compounds were measured in CDCl_3_ using Bruker DRX-600 MHz Ultrashield spectrometers (Bruker BioSpin, Billerica, MA, USA). Isolation and purification of compounds were performed on column chromatography using Diaion HP-20 and silica gel 60 (70–230 mesh, Merck, Darmstadt, Germany). Monitoring of eluted compounds was performed on pre-coated TLC plates with silica gel 60 F_254_ (Merck, Darmstadt, Germany).

### Plant material

Aerial parts of PP were collected from Al-Taif governorate, Saudi Arabia, in April 2015 and identified by staff members of taxonomy at the Faculty of Science & Arts, Khulais, King Abdulaziz University, Kingdom of Saudi Arabia. A Herbarium specimen was dried and kept under code (PP-1065) at the Herbarium of the Faculty of Pharmacy, King Abdulaziz University.

### Extraction and isolation

Three hundred grams of the dried aerial parts of *P*. *punctulata* (PP) was extracted with methanol (2.5 L × 4) using ultra turrax homogenizer at room temperature, until exhaustion and the collected extracts were evaporated under vacuum to give 31 g of brown residue. Total methanol extract (PPT) was suspended in the least amount of water and extracted with chloroform that evaporated under vacuum to give 9 g dark brown residue (fraction I). Three grams of the chloroform fraction were kept for the biological investigation. The aqueous mother liquor was concentrated under vacuum and fractionated on Diaion HP-20 column (6 × 110 cm, 250 g) using water followed by 50% MeOH and 100% MeOH. All collected fractions were evaporated under vacuum to give fractions II, III, and IV, respectively.

The chloroform fraction (6 g) was chromatographed on silica gel column (300 g, 100 × 3 cm) using *n*-hexane with gradual increasing of polarity with EtOAc to obtain eight sub-fractions PP-1 to PP-8. Sub-fraction PP-4 (150 mg) was subjected to silica gel CC (20 g, 50 × 2 cm), and eluted with *n*-hexane:EtOAc (98:2) to afford pure compound **1** (12.1 mg, yellow amorphous powder. Sub-fraction PP-5 (350 mg) was chromatographed on SiO_2_ CC (30 g, 50 × 2 cm), using *n*-hexane:EtOAc (95:5) to give two major compounds that were purified on RP_18_ column using a H_2_O:MeOH (10:90) to give compound **2** (15 mg, yellow amorphous powder) and compound **3** (22 mg, yellow amorphous powder). Sub-fraction PP-6 (200 mg) contained one major spot that was purified on silica gel column (25 g, 50 × 2 cm), using *n*-hexane:EtOAc (90:10) to give compound **4** (10 mg, yellow amorphous powder).

### Aortae isolation

Thoracic aorta was isolated from Wistar rats (6–8 weeks old, King Fahd Medical Research Center, King Abdulaziz University, Jeddah, Saudi Arabia) after they are humanely killed using cervical dislocation, followed by guillotine in order to get rid of blood before clotting. Research Ethics Committee at the Faculty of Pharmacy, King Abdulaziz University approved the study entitled: “Studying the effect of flavonoid from *Psiadia punctulate* on AGEs induced exaggerated vasoconstriction” (# PH-117-39).

### Effect on AGEs induced exaggerated vasoconstriction and impaired dilatation

The vascular reactivity was investigated using the isolated artery technique as described previously [16]. AGEs precursor, methylglyoxal (MG), was used to induce exaggerated vasoconstriction [17] and impaired dilatation as previously described [18]. The isolated aortic rings were left for 20 minutes to acclimatize to the surrounding conditions and reach equilibrium at a 1500 mg ± 50 resting tension. Primary contraction and relaxation of the aorta was then performed by adding phenylephrine (PE, 10^−5^ M) and acetylcholine (Ach, 10^−5^ M) respectively. After the tension was returned to resting state, the AGEs precursor methylglyoxal (MG, 100 μM) was added in the absence or presence of PPT (10–100 μg/ml), different prepared fractions (3–30 μg/ml), or Compounds **1**–**4** (1–10 μM) to the aortic segments and left for incubation at 37°C for 1h. The exposure time was selected based on results of previous publications from our laboratory in which one-hour incubation with MG 100 μM resulted in clear exaggerated vasoconstriction and impaired vasodilation responses [17, 18]. The selected concentrations are those which yielded significant linear vascular results during preliminary experiments. For the control channel, 0.1% DMSO solution was added instead of MG. Following the incubation period, cumulative concentrations of PE and ACh (10^−8^ to 10^−5^ M) were then added.

### Effect on AGEs formation

The effect on AGEs formation was studied as previously reported [12]. In a black 96- well plate, 180 μl of bovine serum albumin (BSA, 10 mg/ml in phosphate buffered saline (PBS)) containing PPT (10–100 μg/ml), bioactive chloroform fraction (Fr. I) (3–30 μg/ml), the isolated Compounds **1–4** (1–10 μg/ml) or aminoguanidine (AG, 1mM), were mixed in the wells. To these mixtures, 20 μl freshly prepared MG (50 mM) was added while 20 μl of PBS was added for the blank. PBS was added instead of MG to a control row of wells containing the same concentrations of PPT, Fr. I or the compounds in order to exclude any autofluorescence (non was significant). The plate was left for incubation at 37°C for one hour in the dark. Fluorescence intensity of the AGEs produced was measured at λex = 325 and λem = 440 nm with the use of Monochromator SpectraMax® M3 plate reader (Molecular Devices, Sunnyvale, CA, USA). Dityrosine and N`-formylkynurenine levels were quantified through fluorescence intensity measurements at λex = 330 and 325 / λem = 415 and 434 respectively [19].

### Free radical scavenging effect

The free radical scavenging activity of PPT, bioactive chloroform fraction, and the isolated Compounds **1–4** was studied as previously reported with some modifications [20]. In a clear 96- well plate, PPT (10–100 μg/ml), Fr. I (3–30 μg/ml), or the compounds (1–10 μg/ml) were incubated with DPPH solution (240 μM) in methanol/tris (1:1 v/v). For the blank; methanol was used instead of PPT, Fr. I or the compounds. DPPH was prepared fresh and used immediately. The vehicle, methanol/tris (1:1 v/v) was added instead of DPPH to a control row of wells containing the same concentrations of PPT, Fr. I or the compounds in order to exclude any auto absorbance (non was significant). The absorbance was measured at 520 nm every minute for 10 minutes with the use of Monochromator SpectraMax® M3 plate reader (Molecular Devices, Sunnyvale, CA, USA).

## Results

### Phytochemical study

**Fig 1:**
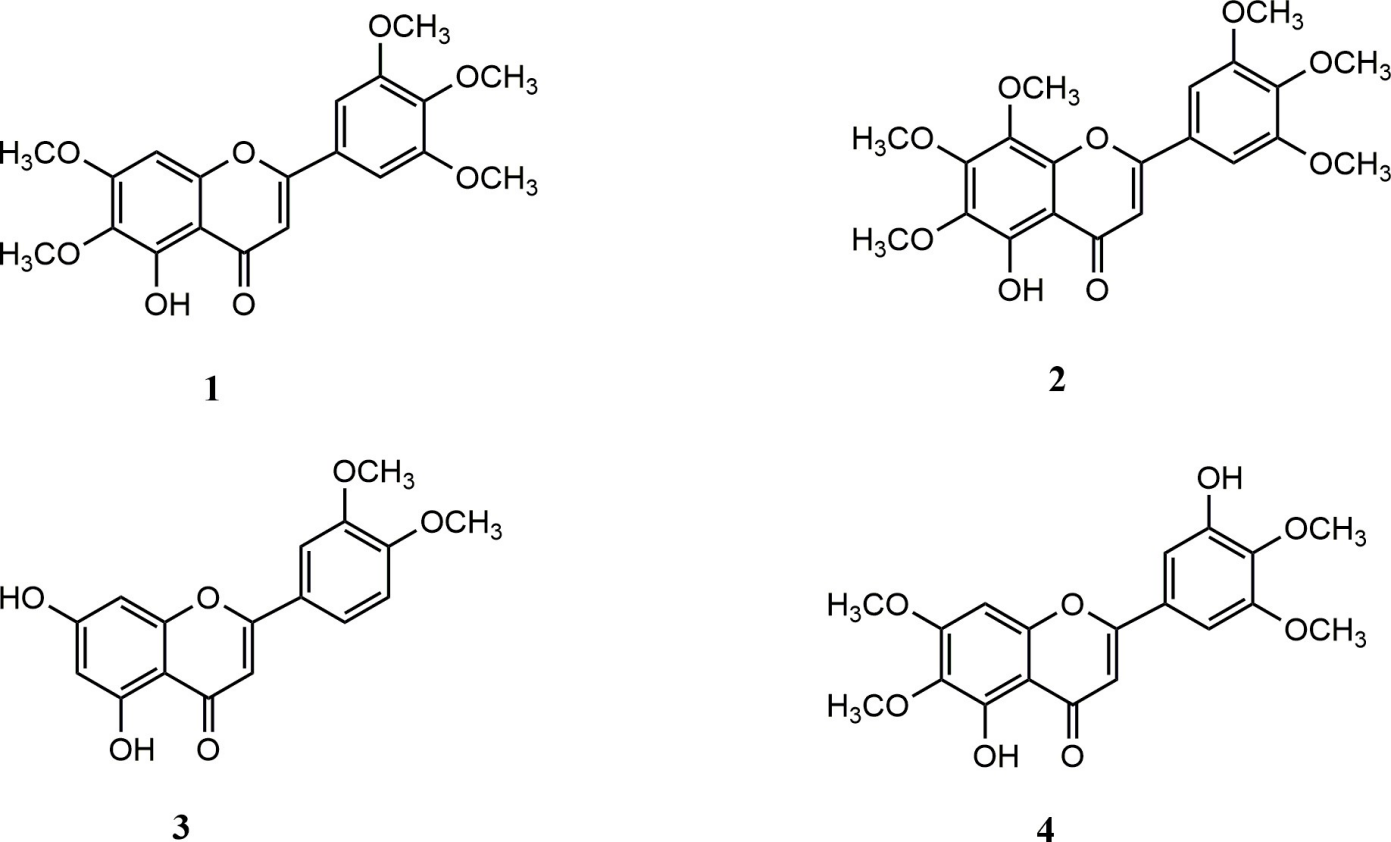
Chemical structures of isolated compounds from *P*. *punctulata*.

Chemical investigation of the bioactive chloroform fraction (Fr I) of *P*. *punctulate* resulted in isolation of four major known phenolic compounds (Fig 1) that were identified using one- and two-dimensions NMR techniques (S1–S10 Figs) as umuhengerin (**1**) [21], gardenin (**2**) [22], luteolin-3`,4`-dimethyl ether (**3**) [23] and 5,3`-dihydroxy-6,7,4`,5`-tetramethoxyflavone (**4**) [24].

### Effect of PPT, bioactive fraction and isolated compounds on AGEs-induced aggravated vasoconstriction

**Fig 2:**
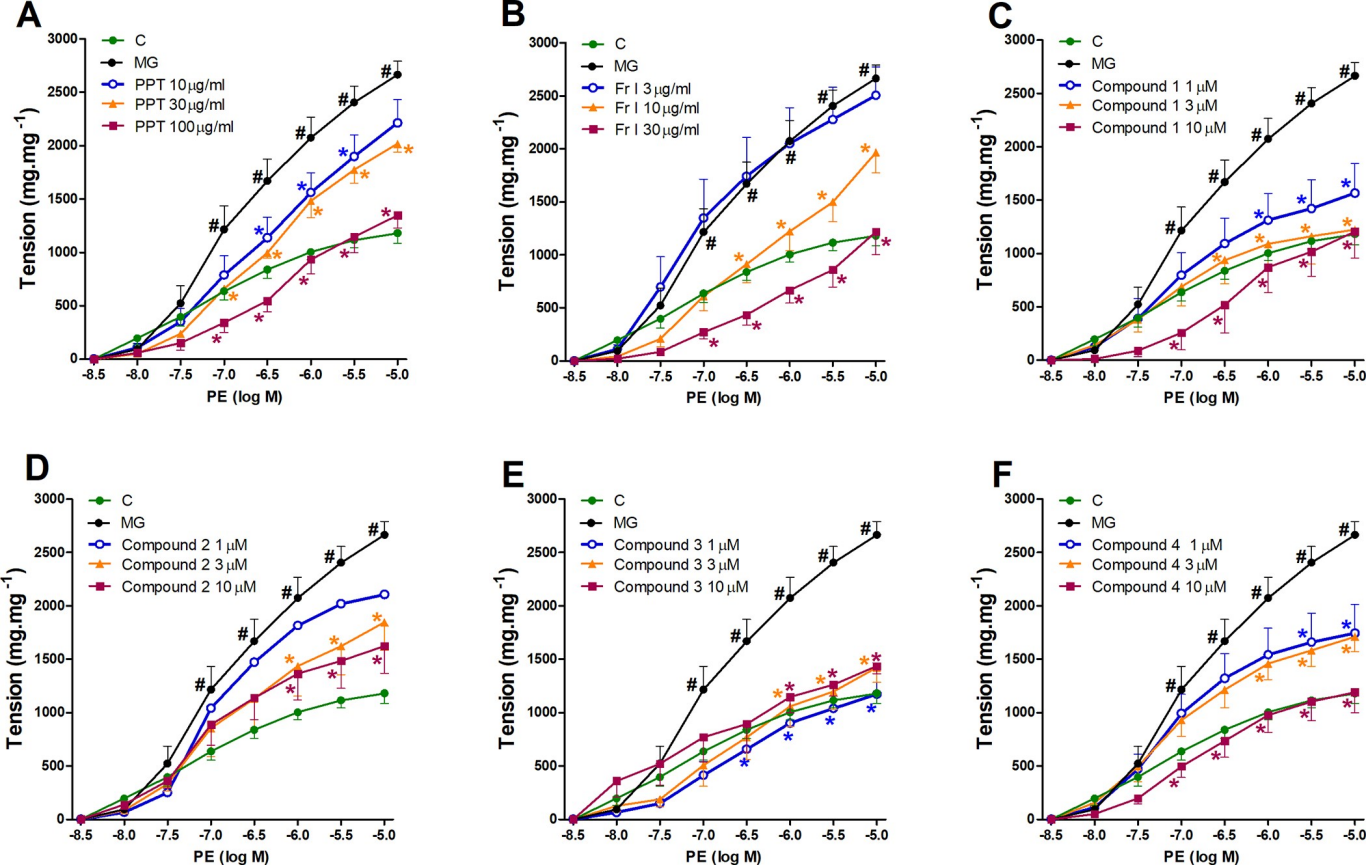
**Effect of one hour incubation of different concentrations of PPT (A), Fr. I (B), and isolated compounds 1–4 (C-F) on the AGEs-induced aggravated responsiveness to PE of aortae isolated from normal rats.** #P < 0.05, compared with the corresponding control values; *P < 0.05, compared with MG group; by Tow Way ANOVA and Bonferroni post hoc test.

Incubation of aortic rings from normal animals with the AGEs precursor, methylglyoxal (MG, 100 μM) for one hour aggravated vasoconstriction responses of aortae to PE (10^−9^ to 10^−5^ M) compared to control group (Fig 2). Incubation of aortic rings for 30 min. with PPT at final concentrations of 10, 30, and 100 μg/mL significantly ameliorated the MG-exaggerated vasoconstriction in a concentration dependent manner (Fig 2A). The bioactive fraction (Fr. I) also produced similar decrease in the MG-exaggerated response at final concentrations of 10 and 30 μg/mL (Fig 2B), therefore, the biological investigations were focused on Fr I and their isolated compounds **1–4**. Compared to MG group, the isolated compounds **1**, **3** and **4** inhibited the exaggerated response at 1, 3 and 10 μM (Fig 2C, 2E and 2F respectively), while compound **2** only showed significant effect at 3 and 10 μM (Fig 2D).

### Effect of PPT, bioactive fraction, and isolated compounds on AGEs-impaired endothelium-dependent vasodilation

**Fig 3:**
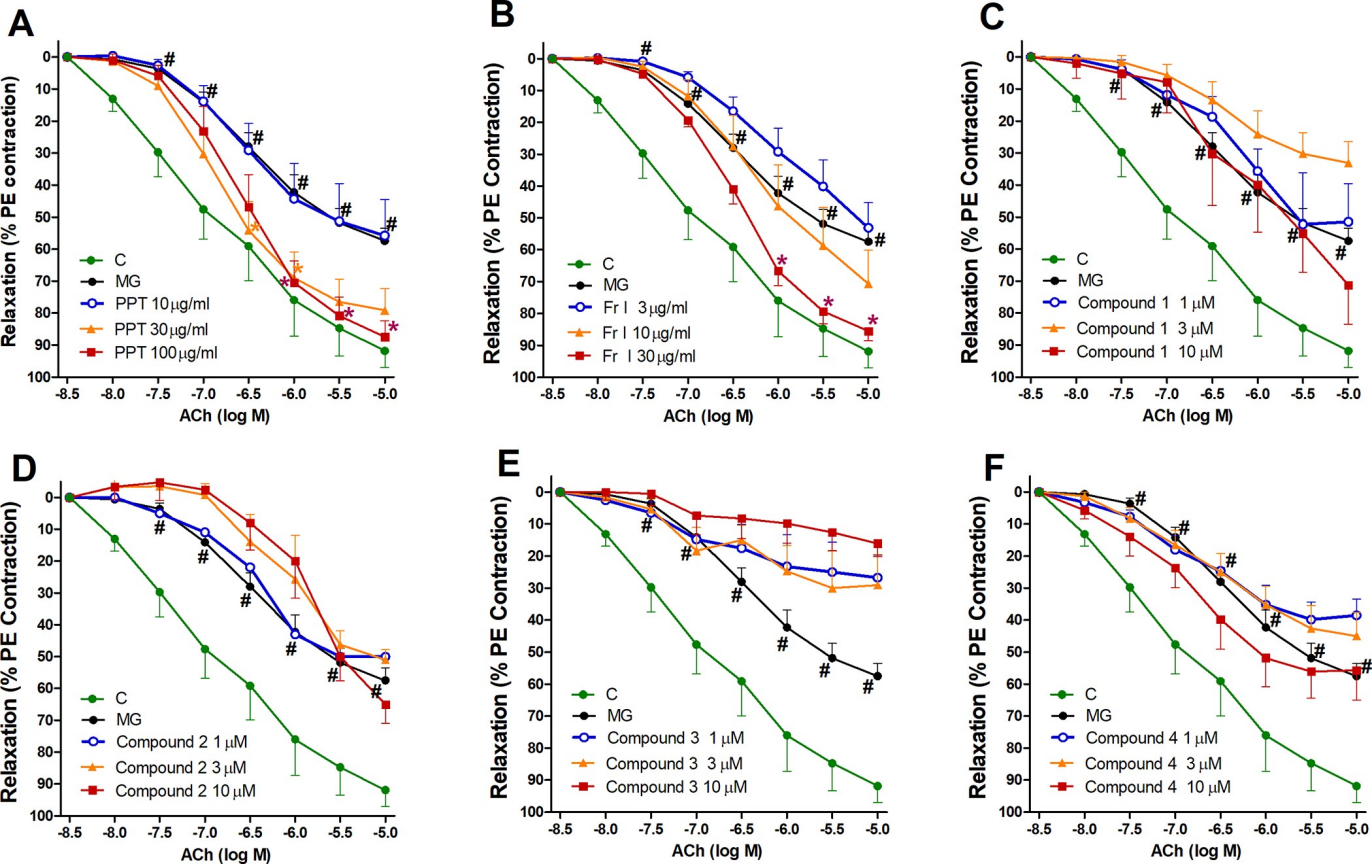
**Effect of one-hour incubation of different concentrations of PPT (A), Fr. I (B), and isolated compounds 1–4 (C-F) on the AGEs-induced impaired vasorelaxant responsiveness to ACh of aortae isolated from normal rats.** #P < 0.05, compared with the corresponding control values; *P < 0.05, compared with MG group; by Tow Way ANOVA and Bonferroni post hoc test.

In comparison with the MG group and among the three tested concentrations, PPT only improved MG-impaired vasorelaxation at the highest concentration of 100 μg/mL (Fig 3A). Fr. I showed similar results (Fig 3B). However, none of the isolated compounds (**1**–**4**) produced any effects on the MG-impaired vasorelaxation at any of the tested concentrations (1, 3, and 10 μM) that inhibited exaggerated vasoconstriction (Fig 3C–3F).

### Effect of PPT, bioactive fraction, and isolated compounds on AGEs formation

**Fig 4:**
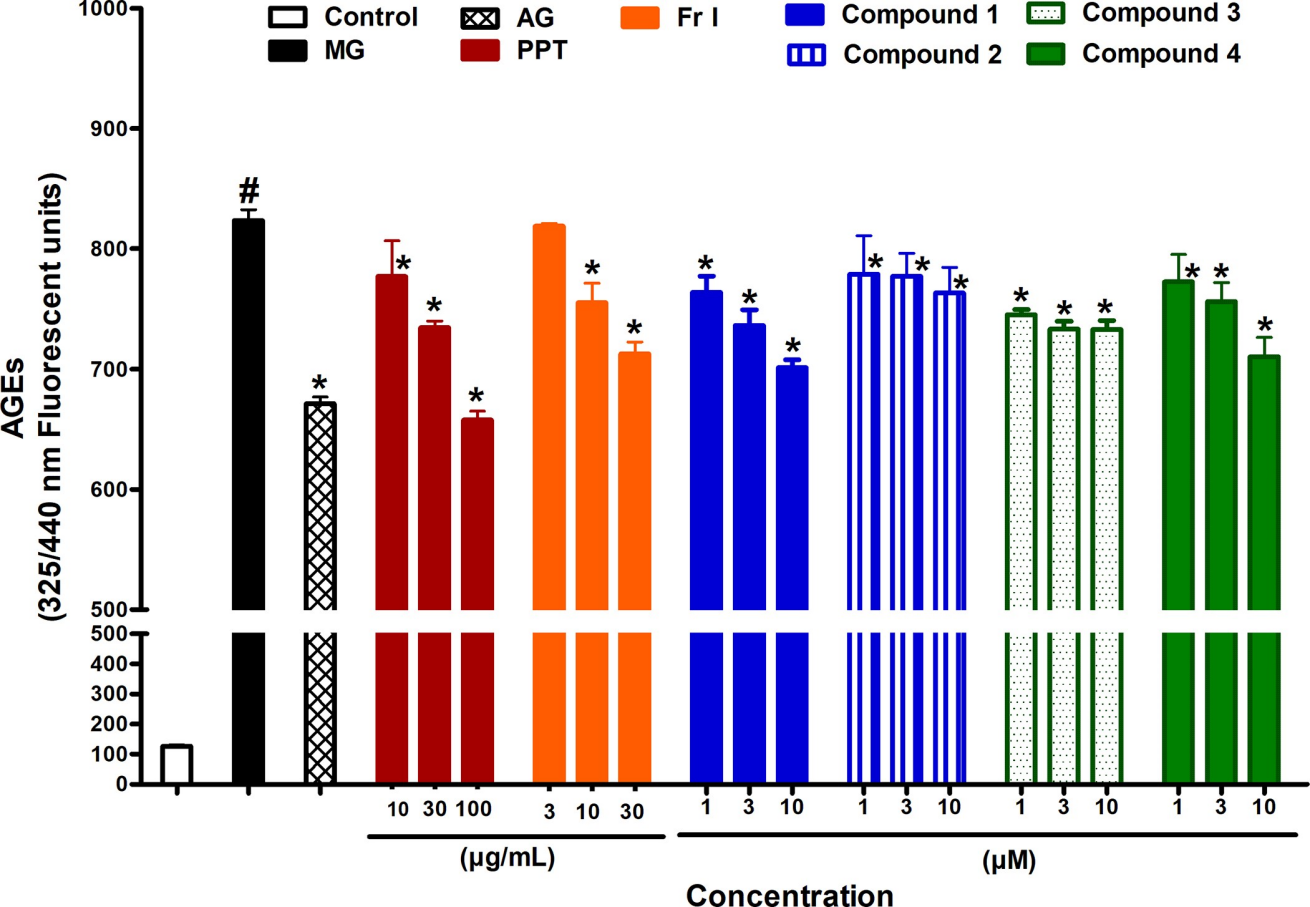
Effect of different concentrations of PPT, bioactive fraction (Fr. I), and isolated compounds 1–4 on the formation of fluorescent AGEs when BSA is incubated with MG for one hour. AG was used as standard anti AGEs drug. Results are expressed as mean ± SEM (n = 3). # p < 0.05 when compared to control group, * p < 0.05 when compared to MG group; by Two Way ANOVA and Bonferroni post hoc test.

Incubation of bovine serum albumin with MG (50 mM) for one hour produced a significant amount of AGEs as indicated from the AGEs-related fluorescence increase. The increase in AGEs fluorescence was significantly inhibited when incubated with the standard AGEs inhibitor, AG (1 mM, as positive control). Incubation with PPT (10, 30 and 100 μg/mL) also significantly inhibited AGEs fluorescence. Similarly, incubation with Fr. I (10 and 30 μg/mL), and the Compounds **1**–**4** (1, 3 and 10 μM) also inhibited AGEs fluorescence compared to MG (Fig 4).

### Effect of PPT, bioactive fraction and isolated compounds on formation of AGEs intermediates

**Fig 5:**
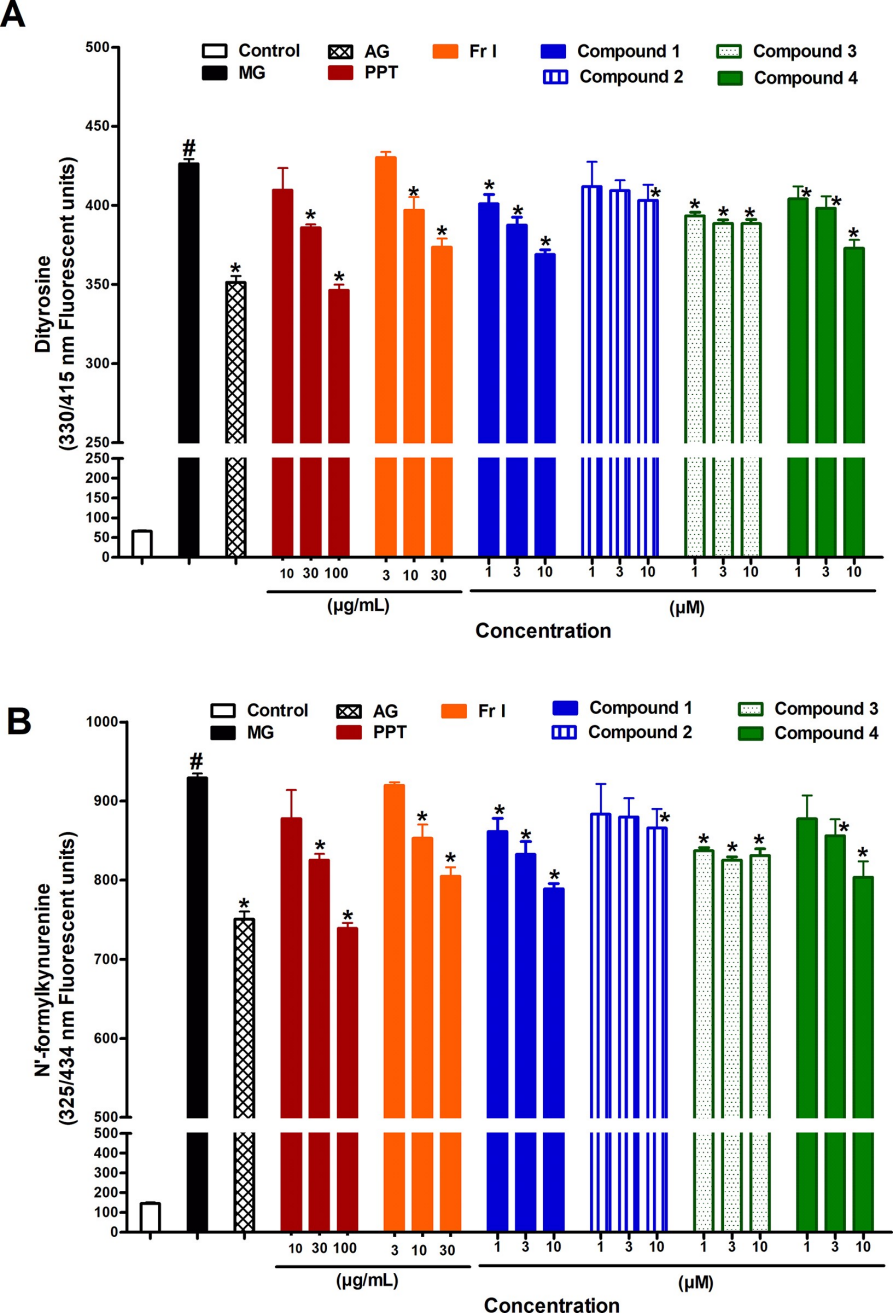
**Effect of different concentrations of PPT, bioactive fraction (Fr. I), and isolated compounds 1–4 on the formation of fluorescent dityrosine (A) and N`-formylkynurenine (NFK), (B) when BSA is incubated with MG for one hour.** AG was used as standard anti AGEs drug. Results are expressed as mean ± SEM (n = 3). # p < 0.05 when compared to control group, * p < 0.05 when compared to MG group; by Two Way ANOVA and Bonferroni post hoc test.

The study measured protein oxidation adducts, particularly dityrosine and N`-formylkynurenine (NFK) as intermediate for AGEs production. MG significantly increased the formation of dityrosine, however addition of PPT (30 and 100 μg/mL) or Fr. I (10 and 30 μg/mL) significantly reduced levels of dityrosine compared to MG group (Fig 5A). Compounds **1, 3** and **4** at all concentration levels, and compound **2 at** 10 μM significantly reduced dityrosine compared to MG group. Similar effects were observed on NFK; another protein oxidation adducts. PPT reduced NFK at concentrations of 30 and 100 μg/mL while Fr. I at 10 and 30 μM/mL. Compounds **1, 3,** and **4 at all concentrations, and Compound 2 at** 10 μM **also** significantly reduced NFK (Fig 5B).

### DDPH free radical scavenging activity of PPT, bioactive fraction and isolated compounds

**Fig 6:**
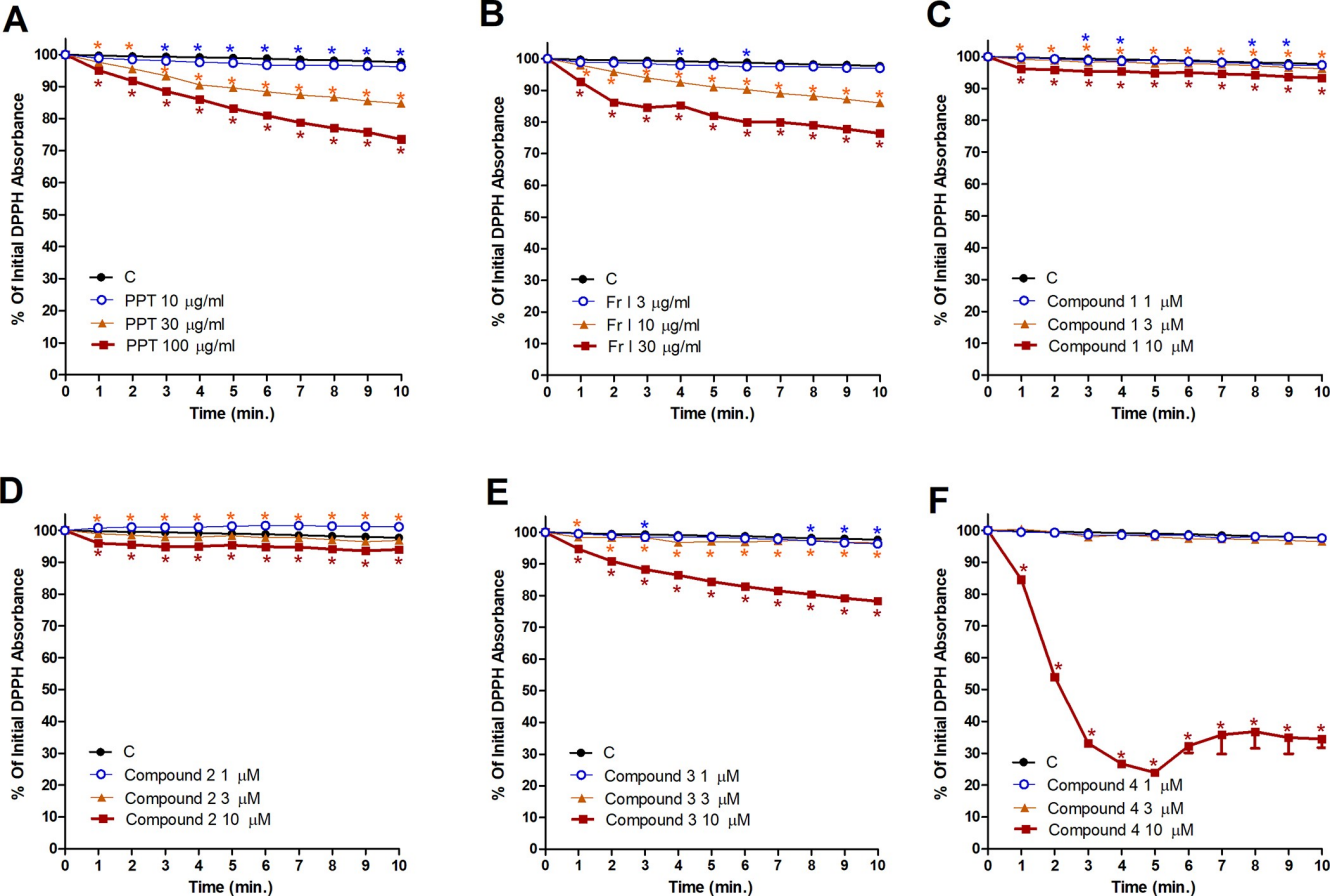
**Free radical scavenging activity of different concentrations of PPT (A), Fr. I (B), and isolated compounds 1–4 (C-F) by the DPPH radical assay.** DPP^●^ radical (violet color) reacts with the extract, bioactive fraction or the isolated compounds to give the reduced form DPPH (pale yellow color) and the intensity of color decreases over time (10 minutes). Data are expressed as percentage decrease of the initial DPPH absorbance. *P < 0.05, compared with the control group; by Tow Way ANOVA and Bonferroni post hoc test.

DPPH assay of PPT showed a concentration-dependent free radical scavenging activity (10% at conc. 30 μg/mL and 21% at conc. 100 μg/mL, Fig 6A). Similar results were obtained with the bioactive fraction Fr. I (8% at conc. 10 μg/mL and 22% at conc. 30 μg/mL, Fig 6B). While, compounds **1** and **2** did not exhibit any radical scavenging activity at any of the three concentration levels (Fig 6C and 6D), compounds **3** and **4** showed marked free radical scavenging activity at concentration of 10 μM (Fig 6E and 6F).

## Discussion

The present study is the first to elucidate the protective effects of *Psiadia punctulata* (PP) on AGEs-induced aggravated vasoconstriction in rat aorta. We designed an integrated study to investigate the pharmacological effects of the methanol extract of PP (PPT), the bioactive chloroform fraction (Fr. I), and the main active compounds (**1–4**) on altered vascular contraction and relaxation, AGEs production, as well as the free radical scavenging activity of the extract and the isolated compounds.

It is well recognized that AGEs aggravate vasoconstriction, which contributes to hypertension in diabetes and metabolic syndrome. In our study, isolated aorta from normal rats showed an overdrawn response to PE when incubated for one hour with MG, a precursor of AGEs production that was in agreement with previous studies [17, 25]. The current study shows that PPT, as well as its chloroform fraction (Fr. I) produced concentration-dependent alleviation of the exaggerated vasoconstriction response to PE, induced by MG. None of the other fractions showed any significant effect. The bioassay-guided fractionation of Fr. I resulted in isolation of four methoxy flavonoids (**1**–**4**) that appear to be responsible for the vascular effects. Flavonoids have special importance in the field of inflammatory and oxidative stress-induced diseases owing to their antioxidative and anti-inflammatory properties. Many flavonoids are known to possess antiglycation properties [26–29], and also known to alleviate AGEs-augmented contractile response to vasoconstrictors in blood vessels [12, 25, 30, 31].

In search for the possible mechanism of the observed vasoconstriction alleviation of PPT, Fr. I and Compounds **1–4**, we examined their effects on vasodilation. While only PPT and the bioactive fraction affected the MG-associated impaired vasodilation at the highest concentrations, none of the isolated compounds **1**–**4** had any significant effect on the impaired vasodilation, while at the same concentrations showing complete alleviation of exaggerated vasoconstriction. This observation excludes the possibility that the alleviation of exaggerated vasoconstriction was mediated via vasodilation.

Inhibition of AGEs formation was also investigated as a possible mechanism of action for the observed vasoconstriction alleviation of PPT, Fr. I and the isolated compounds. MG was used to initiate AGEs formation. MG originates mainly from glucose and fructose metabolism, but can also derive from lipid and amino acid metabolism. Being a very unstable sugar derivative, MG reacts with structural and functional proteins via Millard reaction to form Schiff’s adducts, which then undergo further rearrangement to Amadori products and finally AGEs [32]. AGEs can augment vascular contractility by many receptor-dependent or independent mechanisms[33]. Glycation of the enzymes responsible for antioxidant production increases ROS, which react with and consume NO leading to exaggerated contraction and impaired relaxation [18]. In the present study, the PPT and its bioactive fraction inhibited AGEs production, in a concentration dependent manner. Each of the four isolated compounds also showed similar pharmacologic effect.

The effect on AGEs intermediates was studied in order to explain the observed antiglycation effect of PPT, its bioactive fraction and isolated compounds. Dityrosine is a protein oxidation product comprising two tyrosine residues. The tyrosyl radical is produced as a result of various metabolic processes, including peroxidases-catalyzed reactions, oxidation of hemoglobin, oxidative and nitrative stress, [32] which undergoes further reactions leading to stable irreversibly formed AGEs. Like dityrosine, NFK is an oxidation product of tryptophan formed by oxidation of the indole residue. Both dityrosine and NFK are florescent probes and therefor useful to detect oxidized proteins. The current study showed that PPT, its bioactive fraction and isolated compounds significantly reduced the levels of dityrosine and NFK compared to MG group. This suggests that PPT, its bioactive fraction and isolated compounds interfere with AGEs formation at the early stage of protein oxidation.

To ensure whether the AGE inhibitory effect of the tested compounds was a result of free radical scavenging activity, and whether this plays a role in the observed effects on vascular contraction, free radical scavenging activity was examined using the DPPH method. DPPH assay is widely used for the assessment of the antioxidant capacities of natural products because it measures the radical scavenging ability of antioxidants even when present in complex biological mixtures, such as plant or food extracts. In the current study, the plant extract and the active fraction showed moderate radical scavenging activity. While compound **4** showed strong free radical scavenging activity, compound **3** showed moderate effect, with compounds **1** and **2** showing marginal activity. The free radical scavenging activity could play a role in alleviating exaggerated vasoconstriction as free radicals are known to produce vasoconstriction [17]. In addition, free radical scavenging could inhibit protein glycoxidation and hence AGEs formation.

A number of studies have reported that AGEs formation inhibitory activities of several flavonoids are the result of their DPPH radical scavenging activities [34, 35]. A study of antiglycation activity of 62 flavonoids by Matsuda et al. shows that the number of hydroxyl groups at positions 3ʹ,4 ʹ,5,7, correlate with AGEs-inhibitory activity [36]. In our study, the four isolated compounds share the presence of hydroxyl group at position 5. Also, compound **3** have a second hydroxyl group at position 7, while compound **4** have the second hydroxyl group at position 3ʹ. This could explain why compounds **3** and **4** showed stronger free radical scavenging activity over compounds **1** and **2**. However, upon studying the relation between AGEs-inhibitory activities of flavonoids and their DPPH radical scavenging activities, Matsuda et al. observed that some flavonoids which substantially exhibit AGE formation inhibitory activity, showed weak radical scavenging activities [36], in agreement with the result of our study.

Flavonoids have been shown to inhibit the biosynthesis of AGEs in the early, propagation, and advanced phases of glycation through their antioxidant properties, metal-chelating ability, protein interaction, MG trapping, and/or blocking the receptor for advanced glycation end products (RAGE) [37–39]. The structure/antioxidant activity relationship requires three benzene rings and one hydroxyl group [36]. Based on these observations and our results that show that the four flavones vary in their radical scavenging capacity but are almost equal in their antiglycation effect, as well as their early oxidation intermediates, we suggest that the four isolated flavones directly inhibited AGE biosynthesis probably by one or more of the previously mentioned mechanisms other than free radical scavenging.

Although the four flavones significantly inhibited AGE production, surprisingly, they did not affect the impaired vasodilation associated with AGE. It is well known that endothelium-mediated vasodilation depends primarily on NO, which is partially consumed by AGE-induced oxidative stress. Vasodilation (particularly NO-mediated) is more sensitive than vasoconstriction to small amount of AGEs, and it is likely that the test compounds did not completely block AGEs formation, explaining their lack of effect on vasodilation. Thus, the weak to moderate free radical scavenging activity of the four compounds were not enough to balance the oxidative load generated by AGEs.

In conclusion, PPT alleviates AGEs-induced exaggerated vasoconstriction. The bioassay guided fractions revealed that chloroform fraction and hence compounds **1**–**4** were responsible for the mentioned activities. The protective effect seems to be mediated mainly through their antiglycation activity.

## Supporting information

S1 Fig ^1^HNMR spectra of compound 1. (TIFF)

S2 Fig ^13^CNMR spectra of compound 1. (TIFF)

S3 Fig HSQC spectra of compound 1. (TIFF)

S4 Fig HMBC spectra of compound 1. (TIFF)

S5 Fig ^1^HNMR spectra of compound 2. (TIF)

S6 Fig ^13^CNMR spectra of compound 2. (TIF)

S7 Fig HSQC spectra of compound 2. (TIF)

S8 Fig HMBC spectra of compound 2. (TIFF)

S9 Fig ^1^HNMR spectra of compound 3. (TIFF)

S10 Fig ^13^CNMR spectra of compound 3. (TIFF)

S11 Fig HSQC spectra of compound 3. (TIFF)

S12 Fig HMBC spectra of compound 3. (TIFF)

S13 Fig ^1^HNMR spectra of compound 4. (TIFF)

S14 Fig ^13^CNMR spectra of compound 4. (TIFF)

S15 Fig HSQC spectra of compound 4. (TIFF)

S16 Fig HMBC spectra of compound 4. (TIFF)
