# Supplementary figures and images for: Psiadia punctulata major flavonoids alleviate exaggerated vasoconstriction produced by advanced glycation end products
Source: PLoS One. 2019 Sep 6;14(9):e0222101. doi: 10.1371/journal.pone.0222101 (PMC6730914; doi:10.1371/journal.pone.0222101)

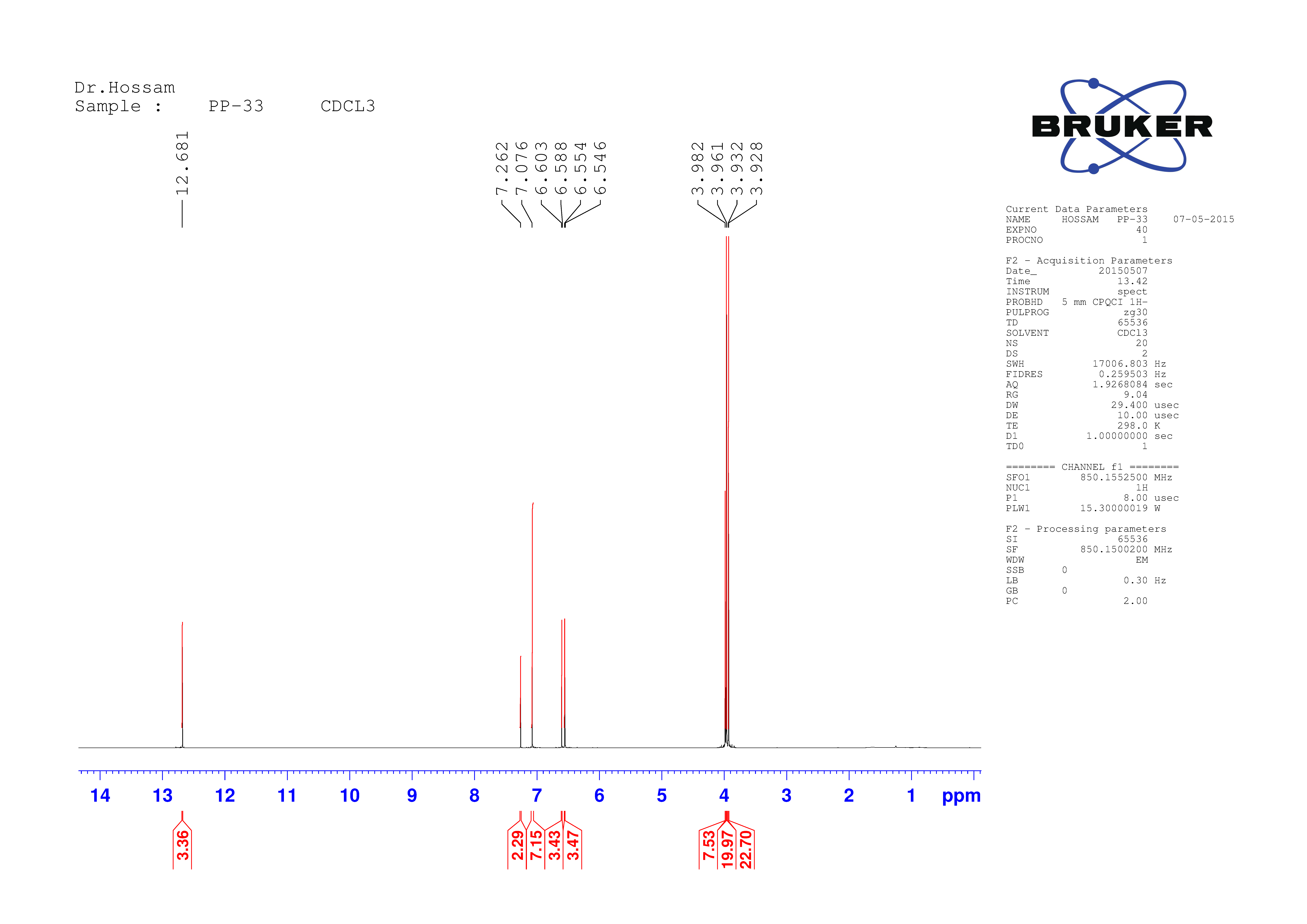

Supplement: S1 Fig — (TIFF) [file pone.0222101.s001.tiff]

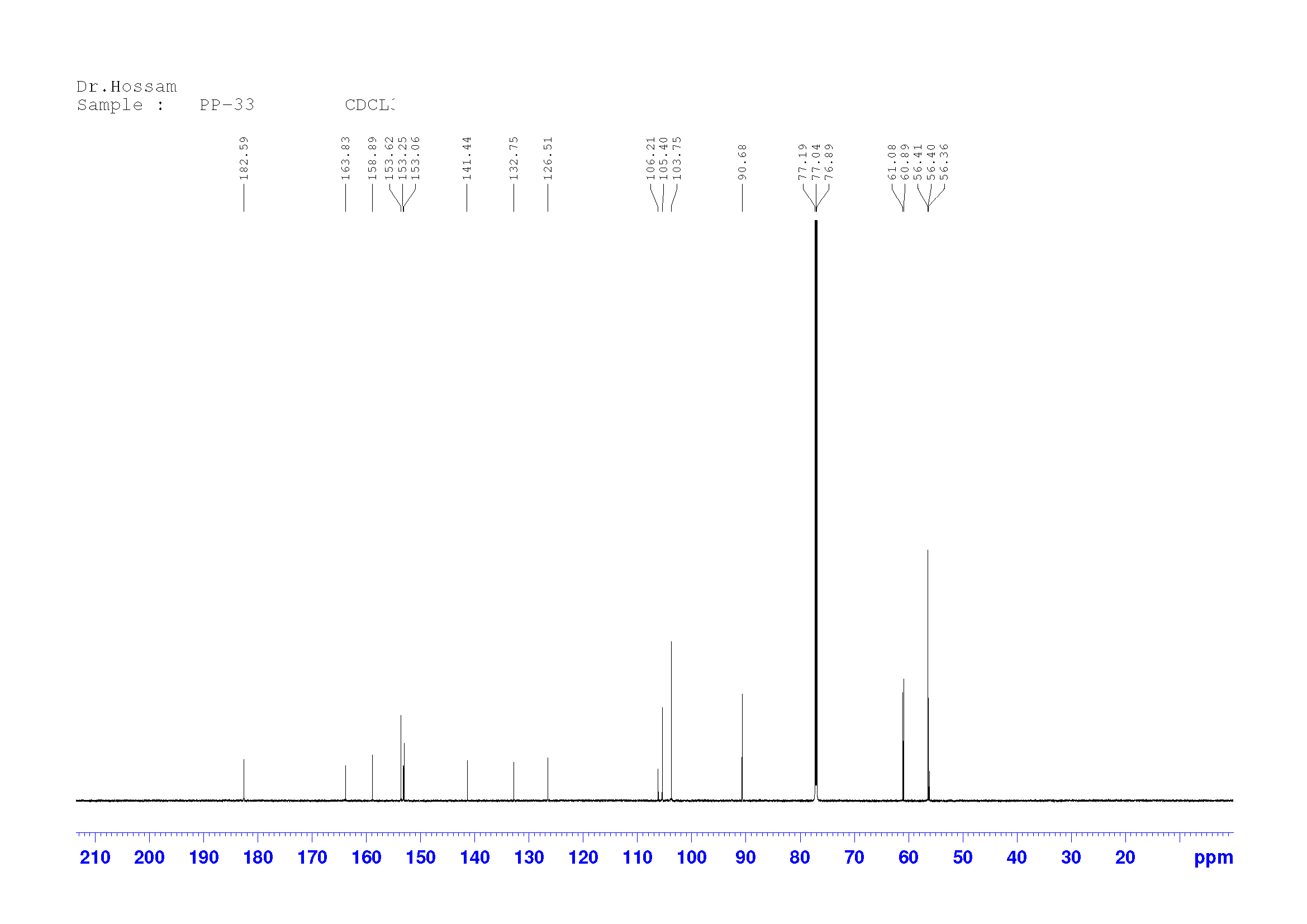

Supplement: S2 Fig — (TIFF) [file pone.0222101.s002.tiff]

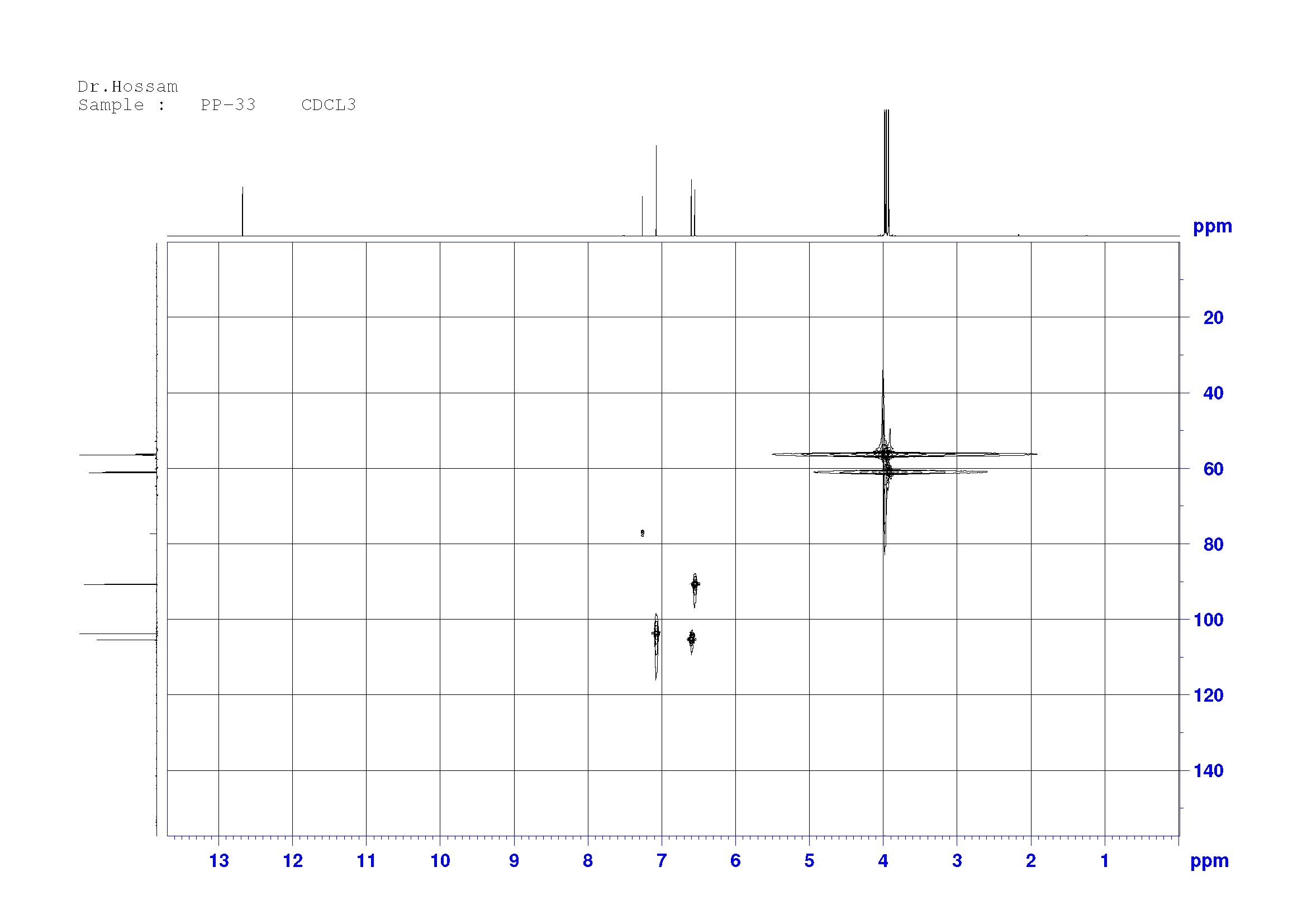

Supplement: S3 Fig — (TIFF) [file pone.0222101.s003.tiff]

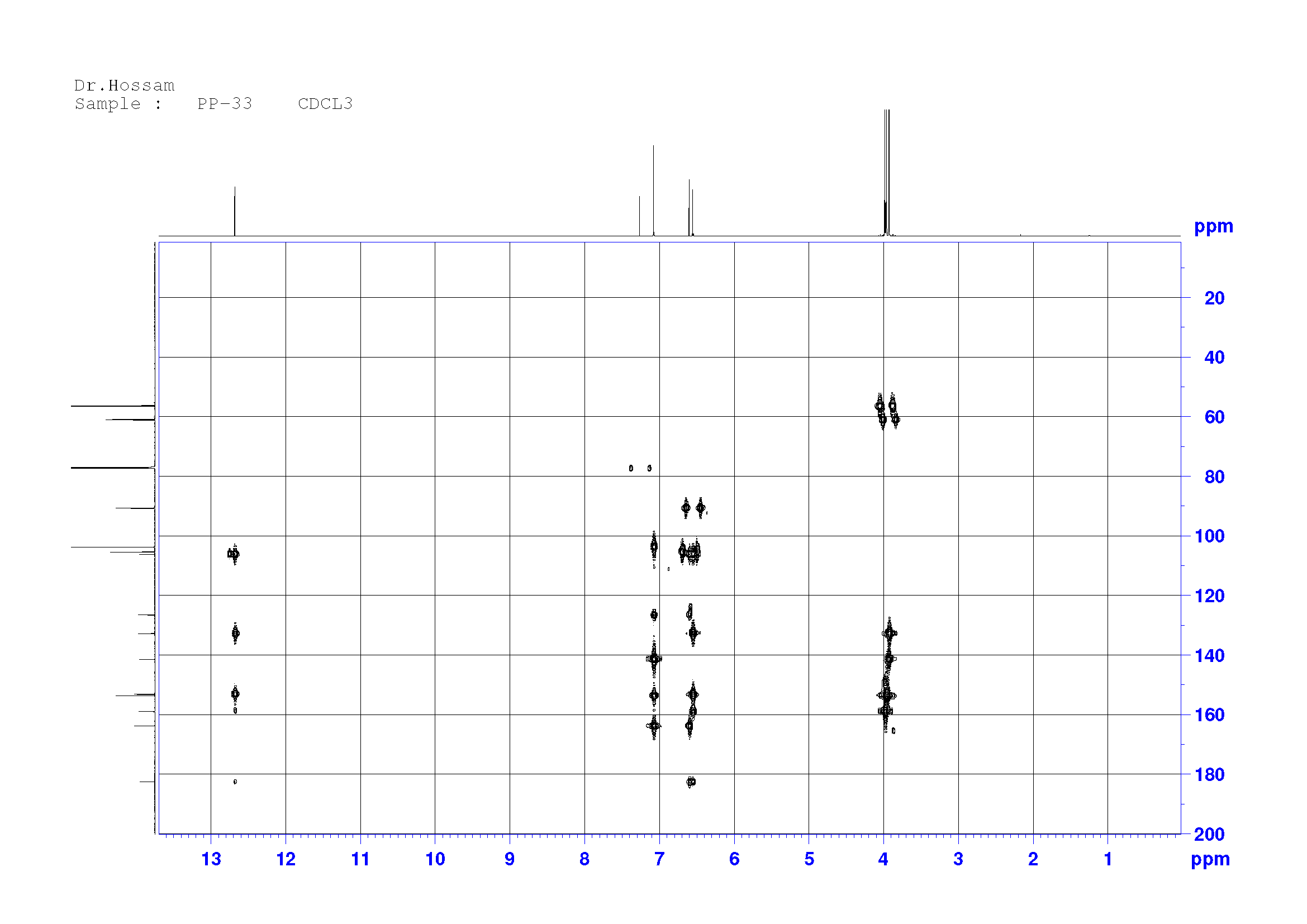

Supplement: S4 Fig — (TIFF) [file pone.0222101.s004.tiff]

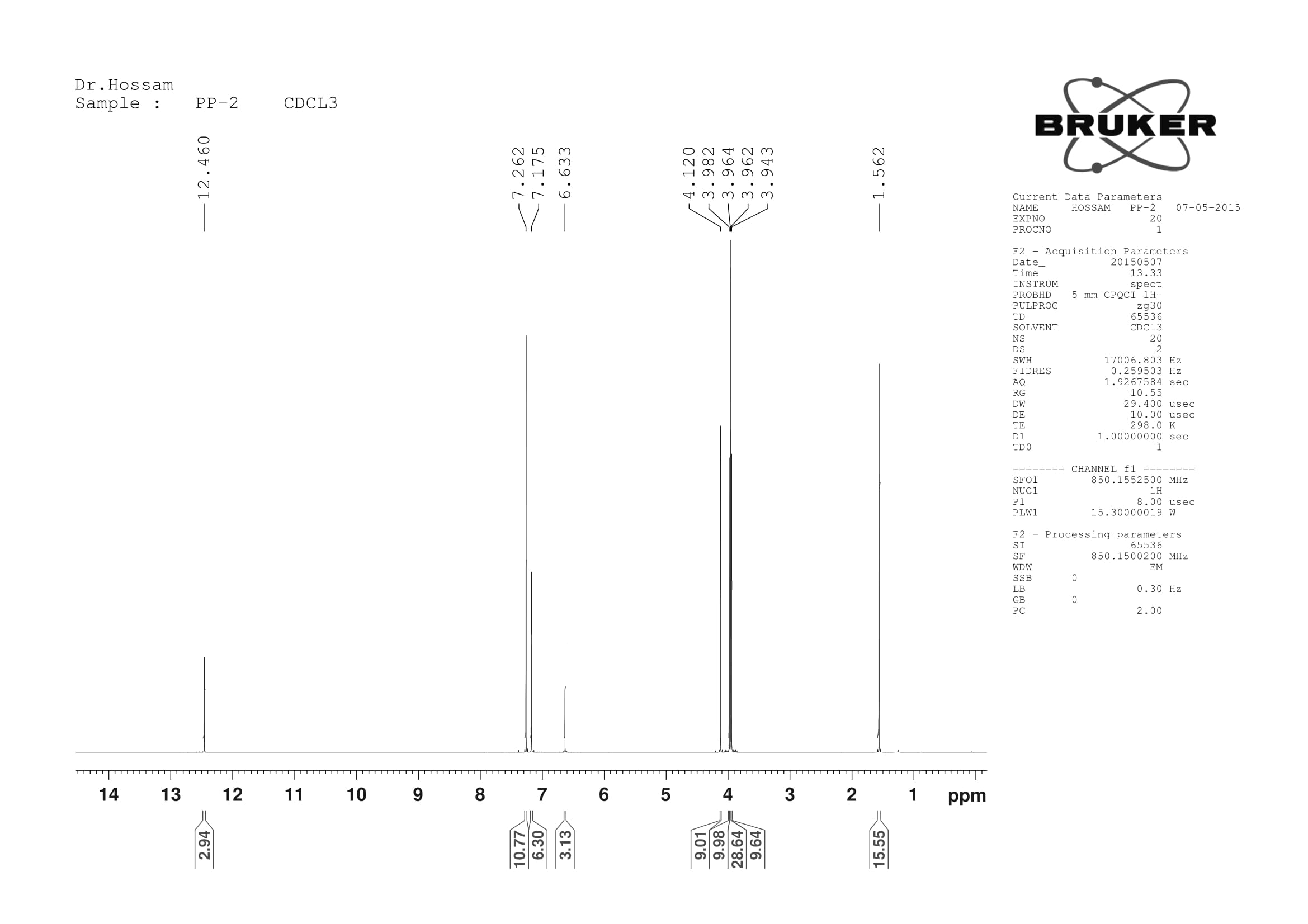

Supplement: S5 Fig — (TIF) [file pone.0222101.s005.tif]

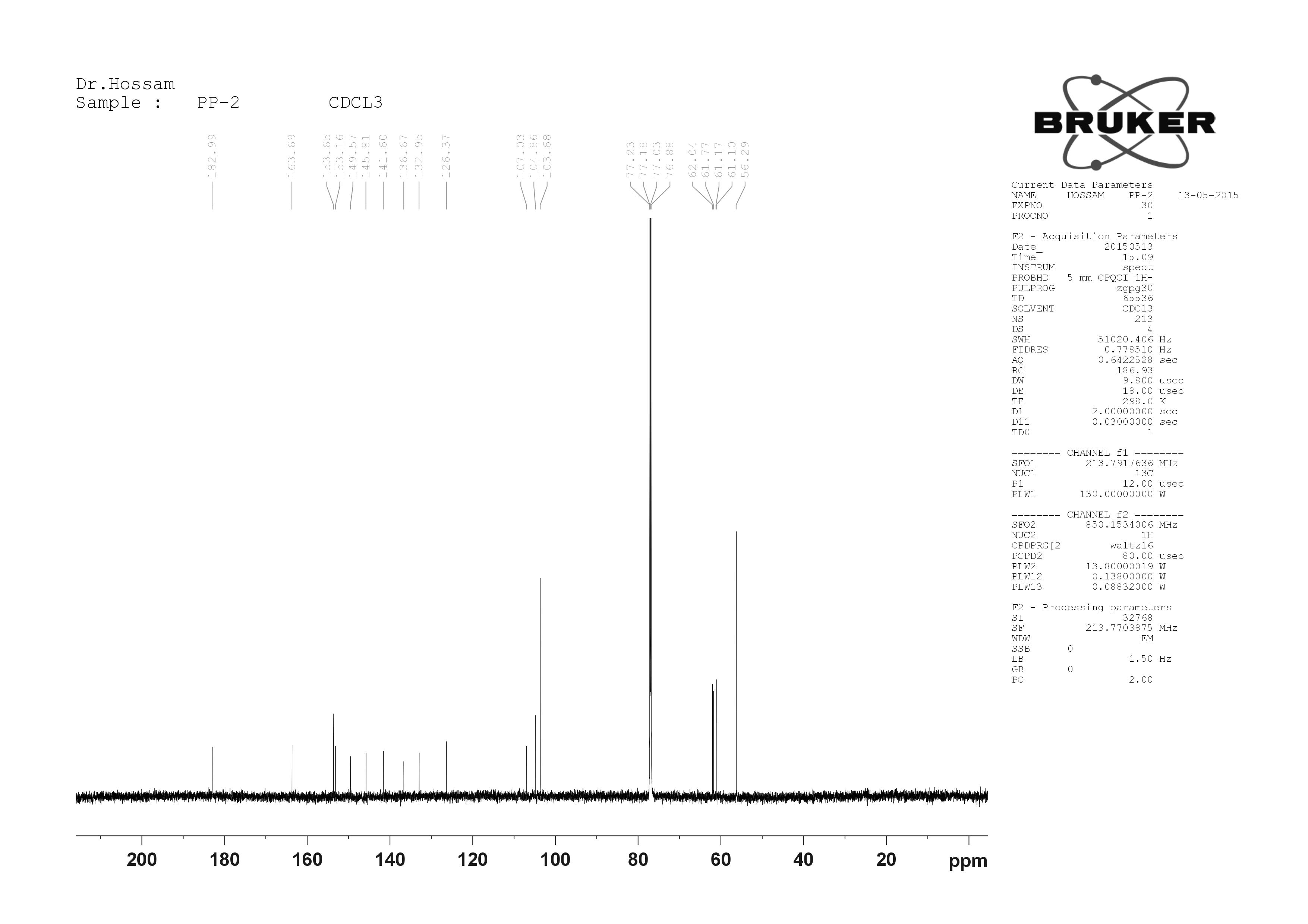

Supplement: S6 Fig — (TIF) [file pone.0222101.s006.tif]

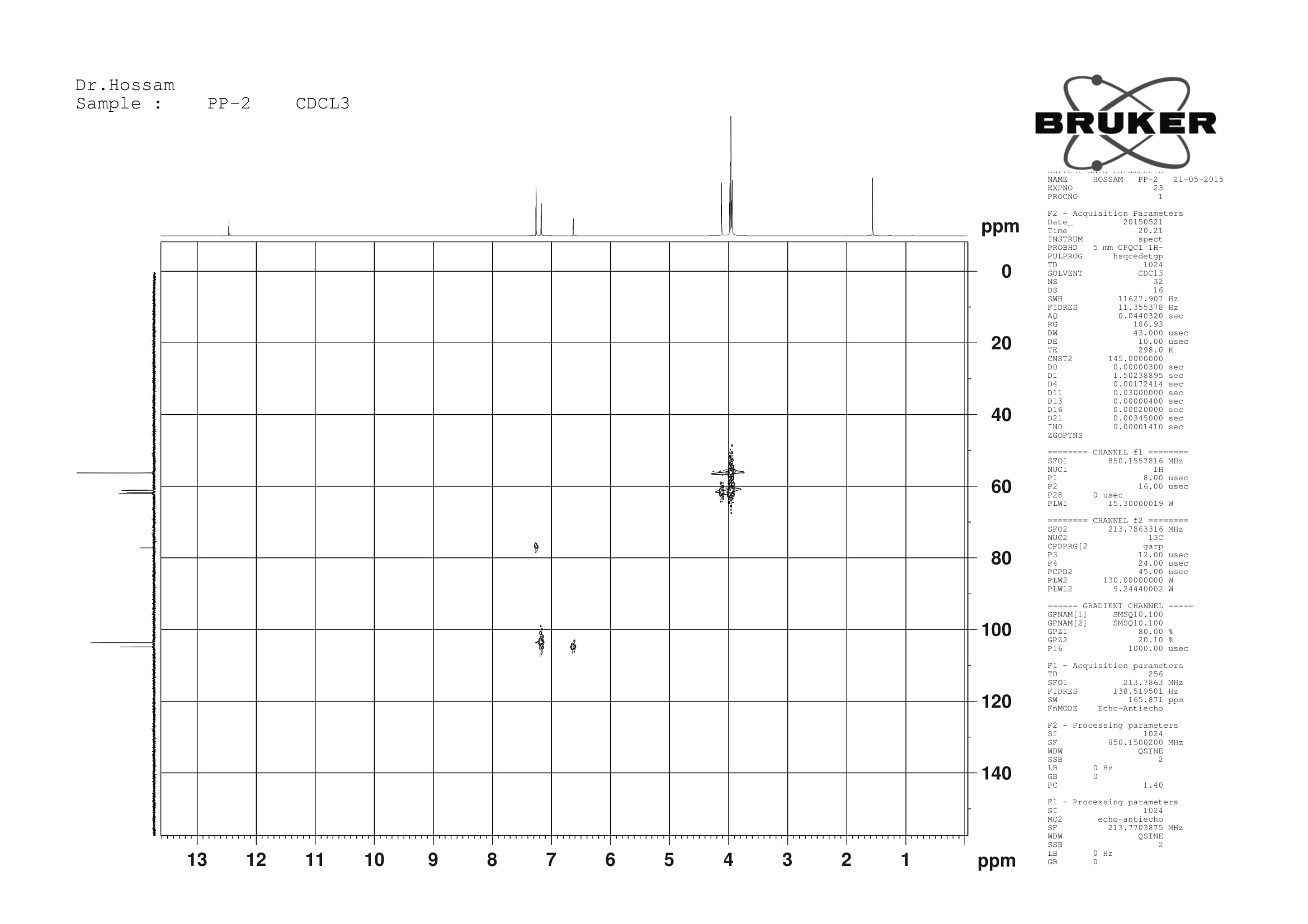

Supplement: S7 Fig — (TIF) [file pone.0222101.s007.tif]

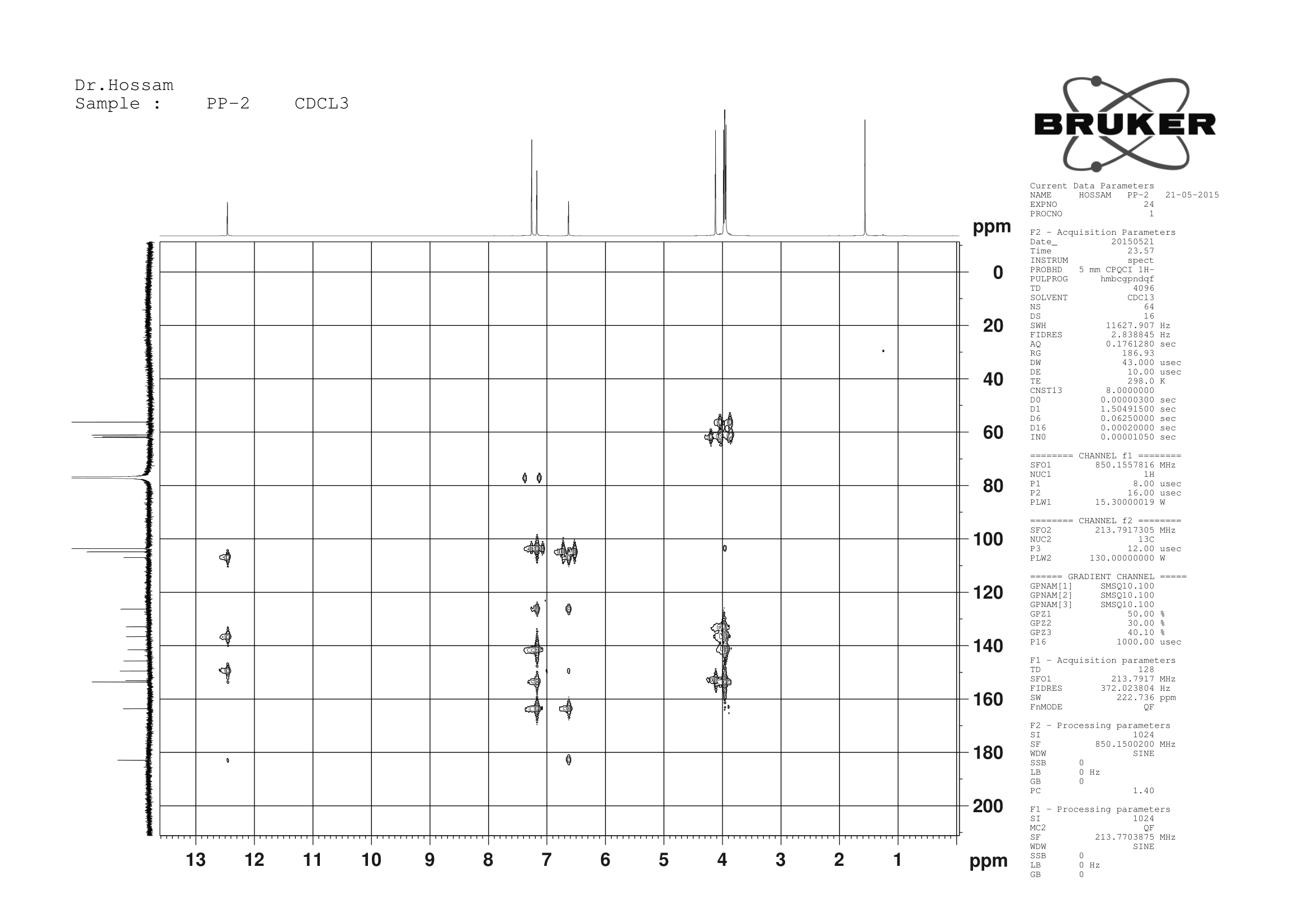

Supplement: S8 Fig — (TIFF) [file pone.0222101.s008.tiff]

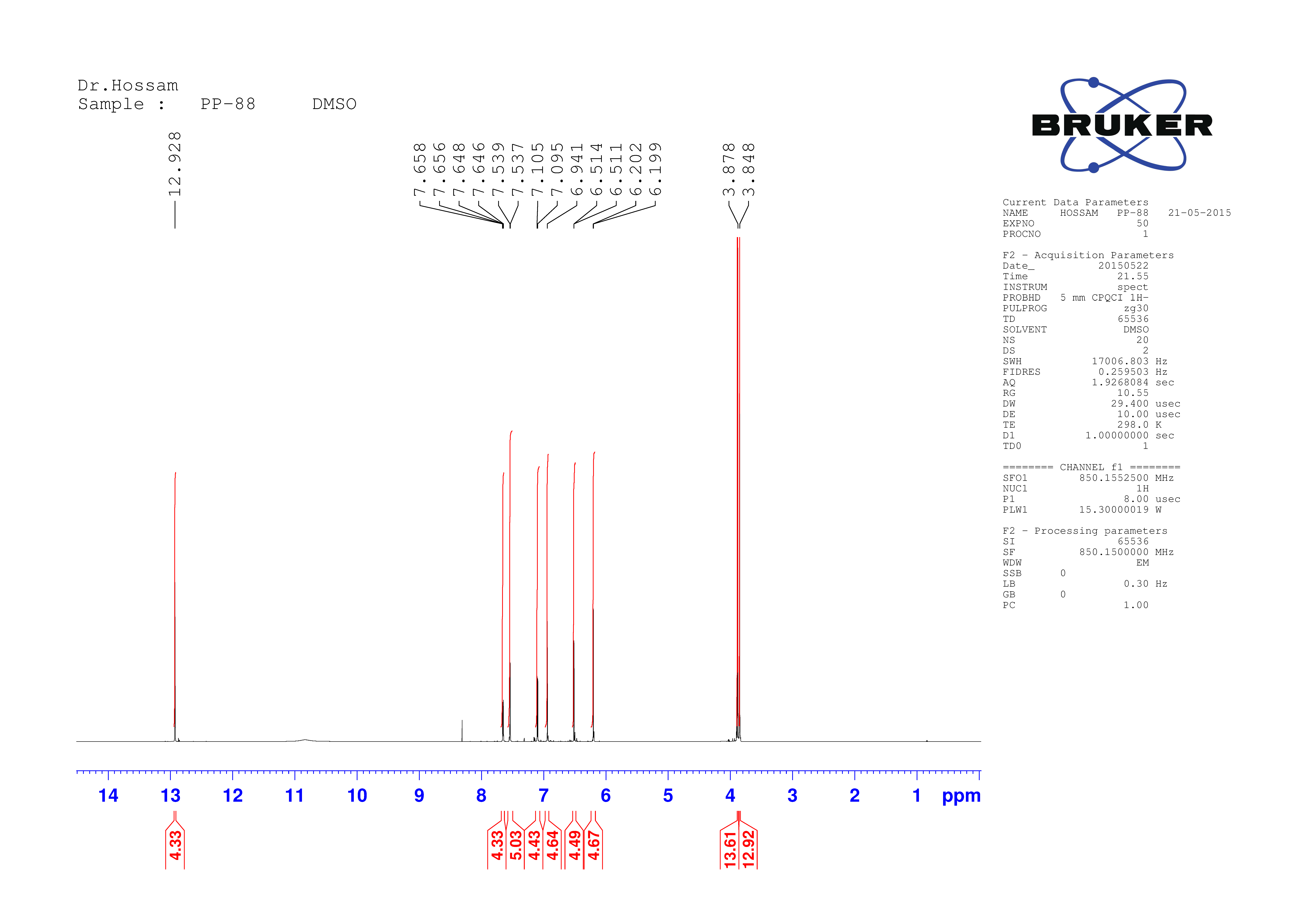

Supplement: S9 Fig — (TIFF) [file pone.0222101.s009.tiff]

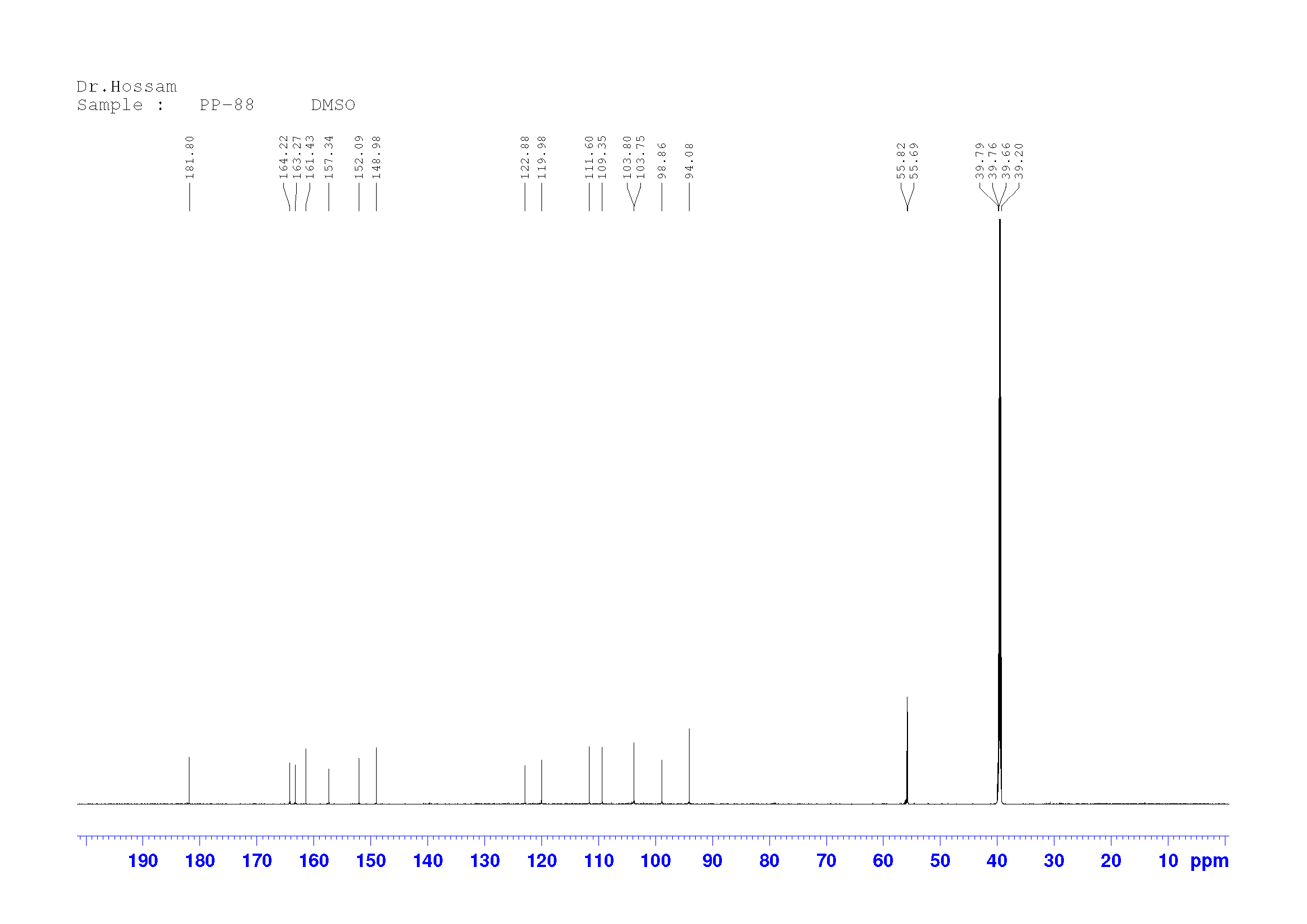

Supplement: S10 Fig — (TIFF) [file pone.0222101.s010.tiff]

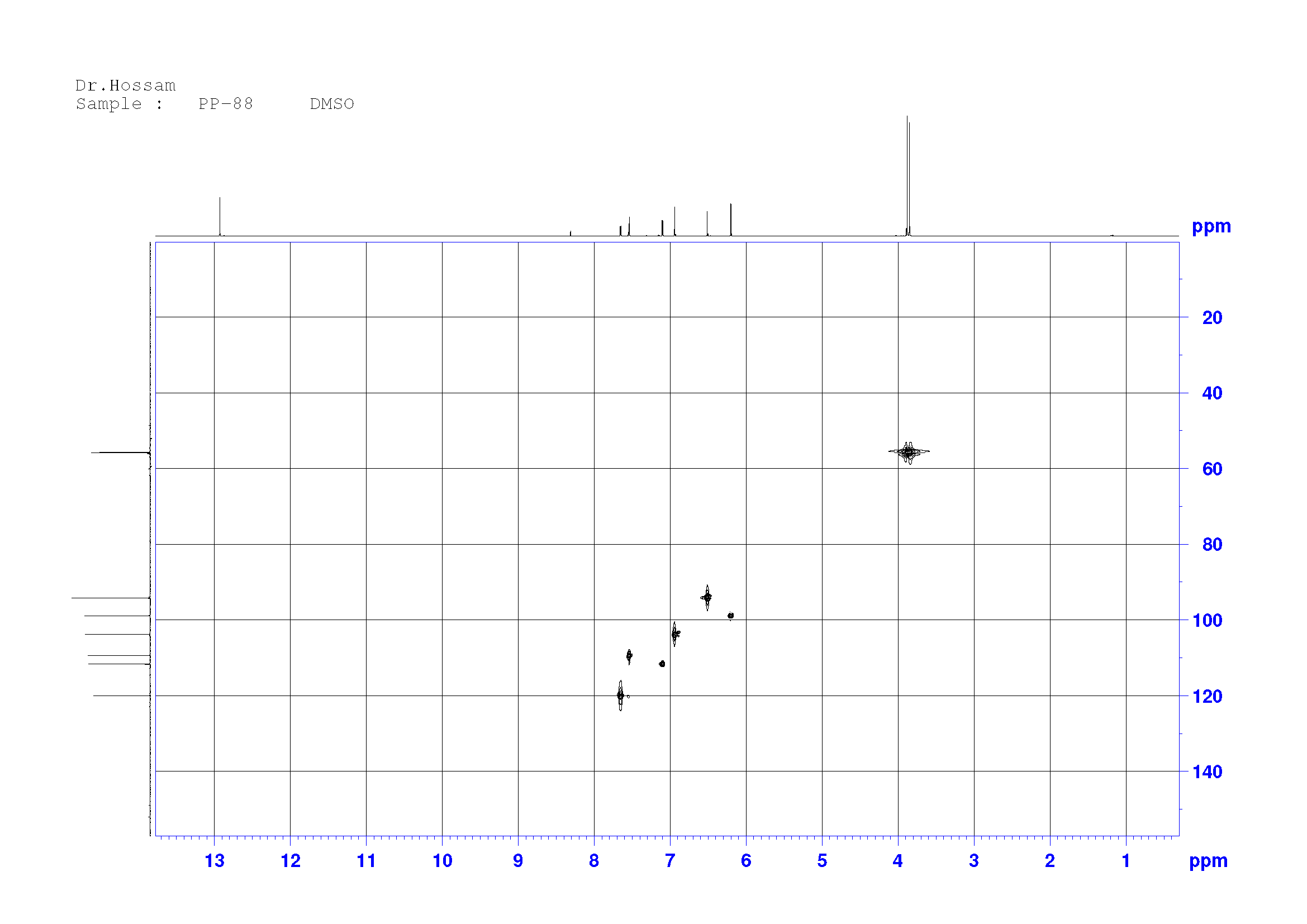

Supplement: S11 Fig — (TIFF) [file pone.0222101.s011.tiff]

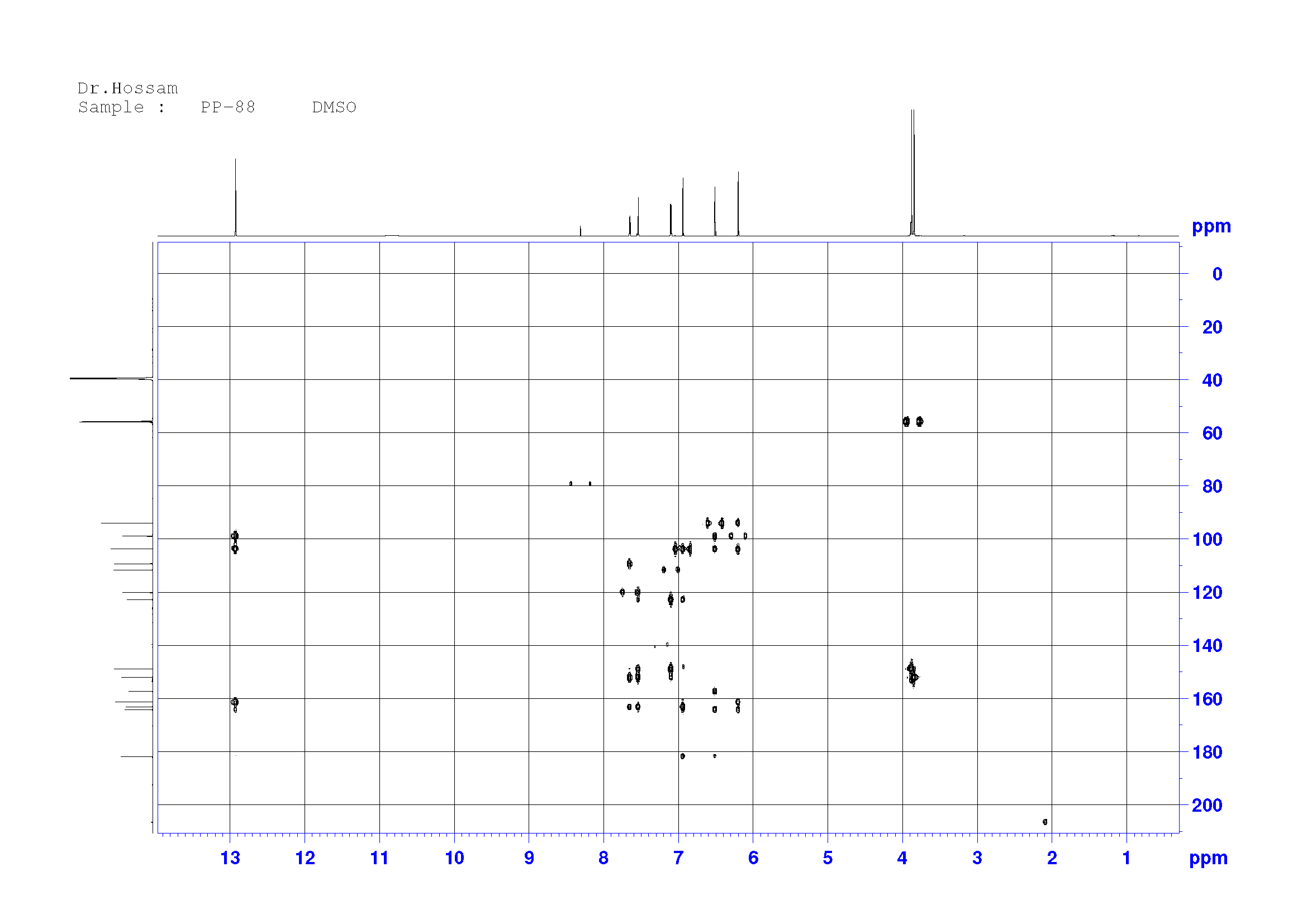

Supplement: S12 Fig — (TIFF) [file pone.0222101.s012.tiff]

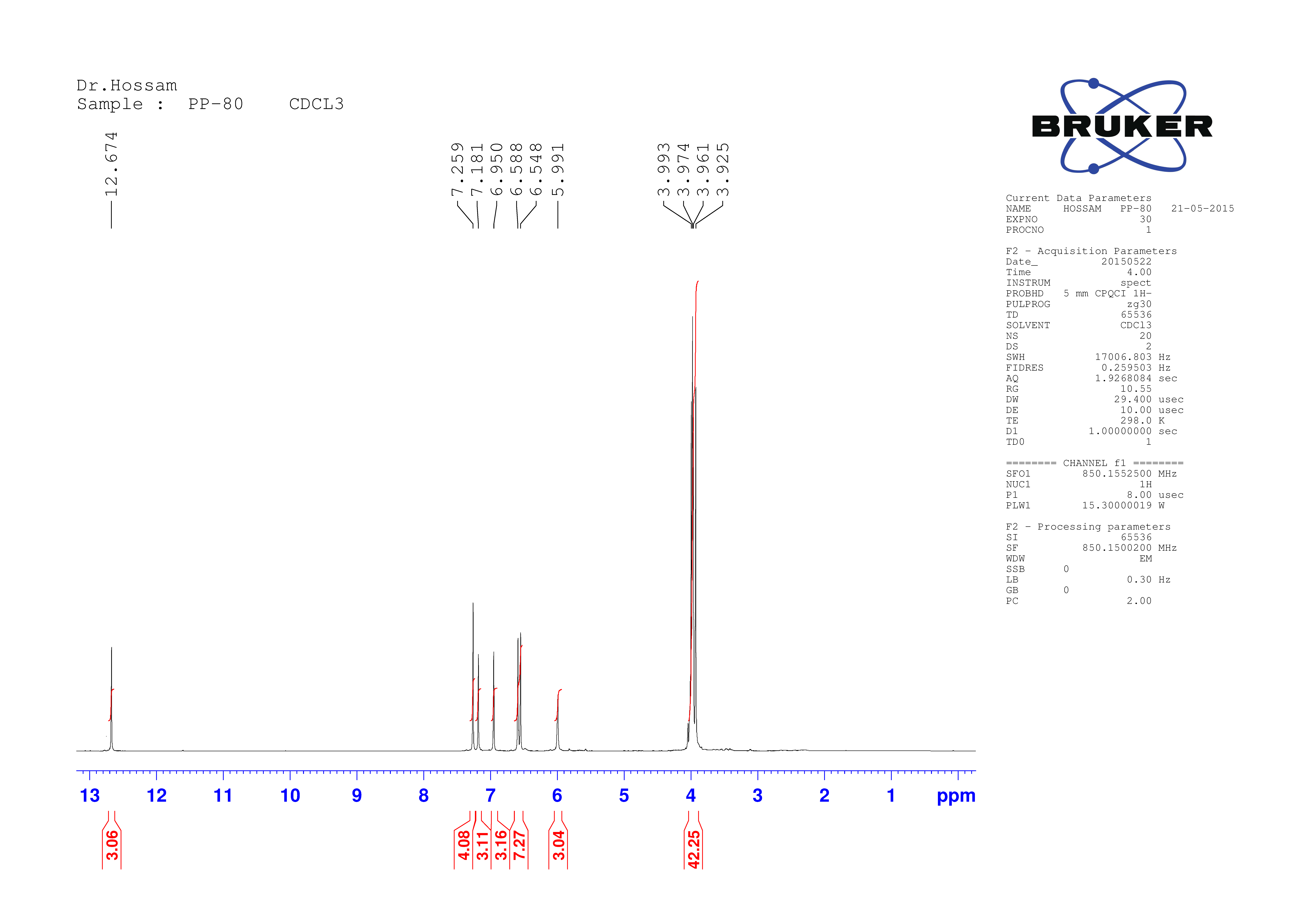

Supplement: S13 Fig — (TIFF) [file pone.0222101.s013.tiff]

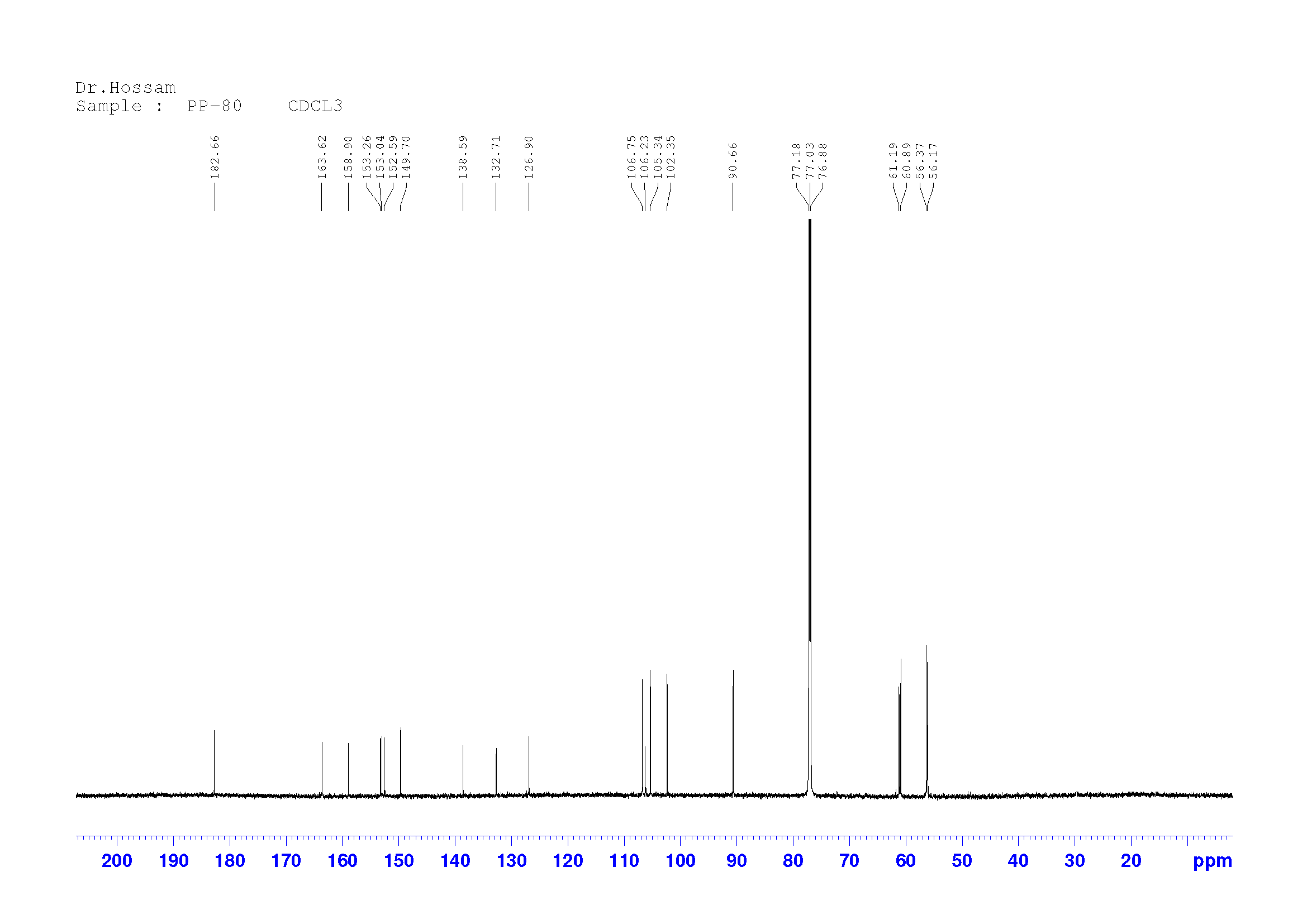

Supplement: S14 Fig — (TIFF) [file pone.0222101.s014.tiff]

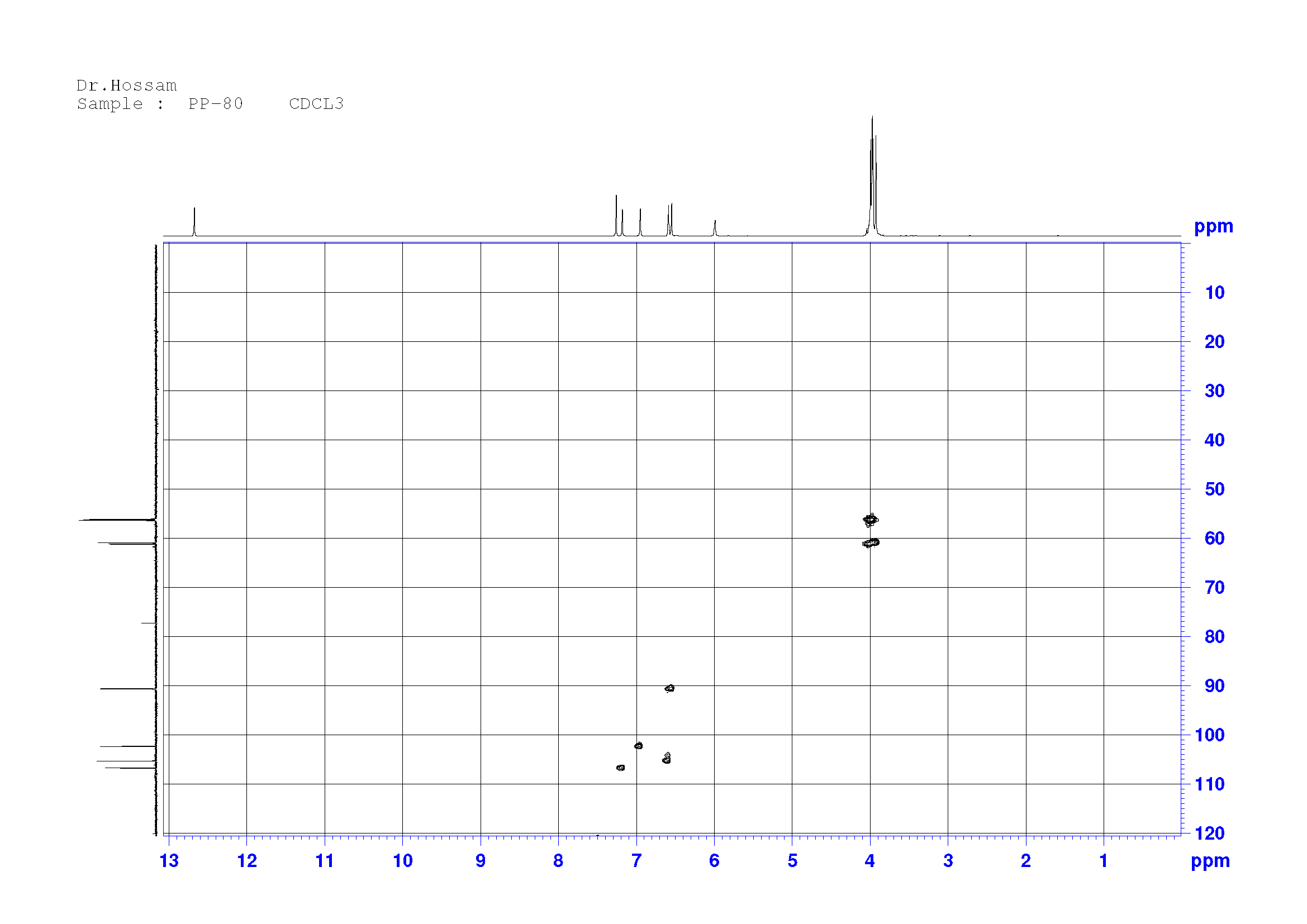

Supplement: S15 Fig — (TIFF) [file pone.0222101.s015.tiff]

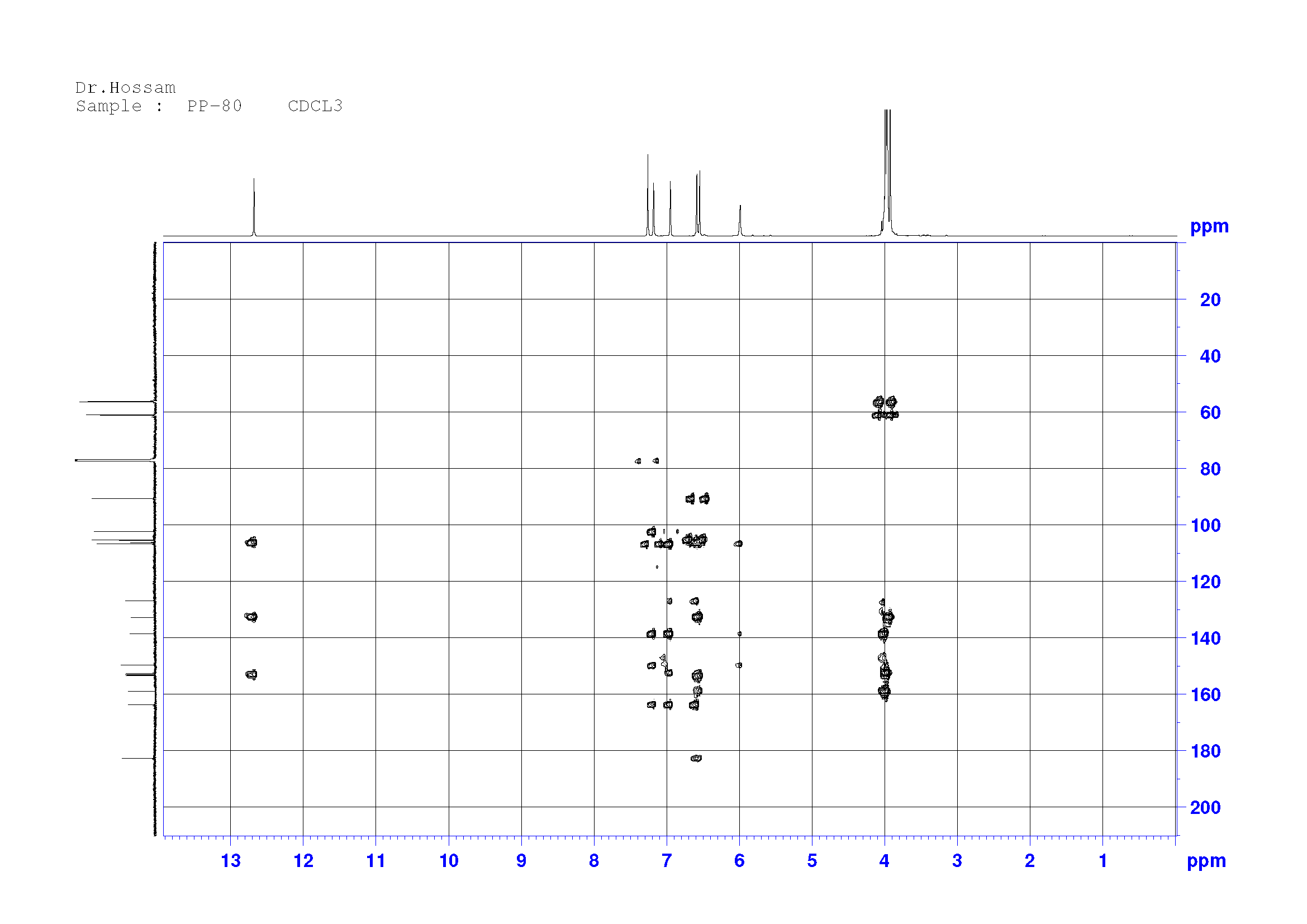

Supplement: S16 Fig — (TIFF) [file pone.0222101.s016.tiff]
